# Proposal for a common nomenclature for fragment ions in mass spectra of lipids

**DOI:** 10.1371/journal.pone.0188394

**Published:** 2017-11-21

**Authors:** Josch K. Pauling, Martin Hermansson, Jürgen Hartler, Klaus Christiansen, Sandra F. Gallego, Bing Peng, Robert Ahrends, Christer S. Ejsing

**Affiliations:** 1 Department of Biochemistry and Molecular Biology, VILLUM Center for Bioanalytical Sciences, University of Southern Denmark, Odense, Denmark; 2 Institute of Computational Biotechnology, Graz University of Technology, Graz, Austria; 3 Omics Center Graz, BioTechMed-Graz, Graz, Austria; 4 Leibniz-Institut für Analytische Wissenschaften-ISAS-e.V., Dortmund, Germany; 5 Cell Biology and Biophysics Unit, European Molecular Biology Laboratory, Heidelberg, Germany; Oregon State University, UNITED STATES

## Abstract

Advances in mass spectrometry-based lipidomics have in recent years prompted efforts to standardize the annotation of the vast number of lipid molecules that can be detected in biological systems. These efforts have focused on cataloguing, naming and drawing chemical structures of intact lipid molecules, but have provided no guidelines for annotation of lipid fragment ions detected using tandem and multi-stage mass spectrometry, albeit these fragment ions are mandatory for structural elucidation and high confidence lipid identification, especially in high throughput lipidomics workflows. Here we propose a nomenclature for the annotation of lipid fragment ions, describe its implementation and present a freely available web application, termed ALEX^123^ lipid calculator, that can be used to query a comprehensive database featuring curated lipid fragmentation information for more than 430,000 potential lipid molecules from 47 lipid classes covering five lipid categories. We note that the nomenclature is generic, extendable to stable isotope-labeled lipid molecules and applicable to automated annotation of fragment ions detected by most contemporary lipidomics platforms, including LC-MS/MS-based routines.

## Introduction

Advances in mass spectrometry (MS)-based lipidomics have enabled comprehensive lipidome analysis at high throughput with generation of large amounts of spectral data that can be harnessed to identify and quantify several hundred lipid molecules in a single sample [1–7]. Applications of this technology have proven useful for both biological and medical sciences by providing mechanistic insights into the regulation of lipid metabolism [8,9], membrane-related processes [10,11], lipid-protein interactions [12–14] and pinpointing lipid biomarkers [15,16]. These advances have also prompted implementation of much needed cheminformatics approaches to classify, catalogue, annotate and depict structures of lipid molecules with complete molecular information about stereochemistry and positions of hydrocarbon chains with locations and configurations of double bonds, hydroxyl groups or other functional groups [17–19]. However, lipidomics technology, even when combined with liquid chromatography (LC) or other separation techniques, is rarely able to provide spectral information that allows the exact structure of a lipid molecule to be determined. To address this discrepancy guidelines have recently been issued to use a hierarchical nomenclature system that annotates lipid molecules with an appropriate shorthand notation that matches the structural information provided by the applied lipidomics technology [20,21]. These guidelines, however, focus only on the naming of intact lipid molecules and not on the underlying lipid fragment ions that are mandatory for structural elucidation and high confidence lipid identification. Notably, this strongly contrasts the conventions put forward in the field of proteomics where a consensus nomenclature to annotate peptide fragment ions and elucidate their amino acid sequence has been in effect for more than three decades [22,23].

Structural characterization of lipid molecules is most commonly performed by tandem MS analysis (termed MS^2^ or MS/MS) where an intact lipid precursor ion is isolated by a mass analyzer, subjected to collision-induced dissociation (CID) and generated fragment ions are subsequently detected using either a low or a high mass resolution detector system [24–28]. Furthermore, mass spectrometers with ion trapping capabilities support multi-stage activation (MS^n≥3^) where fragment ions can be subjected to additional rounds of CID for in-depth structural analysis [1,29,30]. Mechanistic studies of lipid fragmentation pathways have been carried out for a wide range of lipid molecules, including fatty acyls (FAs), glycerolipids, glycerophospholipids, sphingolipids and sterol lipids (reviewed in [31–36]). These studies have shown that CID of lipid molecules occurs via two predominant mechanisms, namely charge-mediated processes that involve the charge of the precursor ion and charge-remote processes that take place physically remote from the charge site. These fragmentation mechanisms yield common and predictable fragment ions from lipid molecules having diverse chemical structures. For example, CID of formate adducts of phosphatidylcholine (PC), lysophosphatidylcholine (LPC), ether-linked phosphatidylcholine (PC O-) and sphingomyelin (SM) in negative ion mode yields a common loss of 60.0211 Da, corresponding to charge-mediated loss of methyl formate (where the methyl group is derived from the choline residue) [24,29]. CID of these lipids also yield fragment ions attributed to the loss of methyl formate combined with charge-mediated neutral loss of FA moieties as ketenes and charge-remote loss of FA moieties as fatty acids (except for SM). Analogously, CID of ammonium adducts of triacylglycerol (TAG), diacylglycerol (DAG), phosphatidic acid (PA) and steryl ester (SE) in positive ion mode yields fragment ions matching the loss of 17.0266 Da (i.e. loss of ammonia) combined with charge-remote loss of FA moieties as fatty acids. Notably, despite these commonalities in fragmentation pathways there is still no consensus nomenclature for shorthand notation of lipid fragment ions.

Annotation of mass spectra of intact precursor ions is based on the tradition that uncharged molecules are represented by the symbol M and that charged derivatives corresponding to loss or gain of a proton are denoted as [M-H]^-^ or [M+H]^+^, respectively. Similarly, association with positive and negative adduct ions such as sodium and formate are denoted as [M+Na]^+^ and [M+HCOO]^-^, respectively. For lipids this convention is often adapted by substituting M with shorthand notation for intact lipid molecules (e.g. [PC 34:1+H]^+^). In comparison, the annotation of lipid fragment ions is much more inconsistently implemented and ranges from graphical displays of complex MS^n^ spectra without any shorthand notation to use of chemical formulas and nominal masses to signify structures of fragment ions (e.g. C_5_H_15_NO_4_P can be used to indicate *m/z* 184.0733 released from phosphocholine-containing lipids; [M-15]^-^ is typically used to indicate loss of a methyl group from choline-containing lipids). While chemical formulas can serve as unique identifiers of fragment ions and nominal masses can help indicate fragmentation pathways, these shorthand notations often make it unintuitive and complicated to relate fragment ions back to the structure of the intact lipid molecule. A more informative approach is to denote fragment ions in reference to structural attributes of the intact lipid molecule. For example, many glycerophospholipids, including phosphatidylethanolamine (PE) and phosphatidylserine (PS), lose their head groups to yield fragment ions with structures that resemble “biological” lipids such as DAG and PA, that in turn can be denoted using shorthand notation resembling intact lipids (e.g. [DAG 36:1+H-18]^+^ and [PA 38:4-H]^-^). Similarly, fragmentation of many lipids yield FA carboxylate anions analogous to deprotonated non-esterified fatty acids (e.g. [NEFA 16:0-H]^-^). However, using names of intact lipids to annotate lipid fragment ions is misleading as, for example, FA carboxylate fragment ions are not produced by simple deprotonation but instead derive from a charge-mediated fragmentation process. Notably, implementation of nomenclature for lipid fragment ions could help novice and experienced lipidomists to more easily read fragment ion spectra and assess the merits of lipid identifications. Moreover, it would also help to implement reporting standards that secure the quality of high throughput lipidomics resource data that is being produced at an accelerated rate nowadays [21,37]. In addition, this would also help curate fragment ion information in databases and search engines.

**Fig 1:**
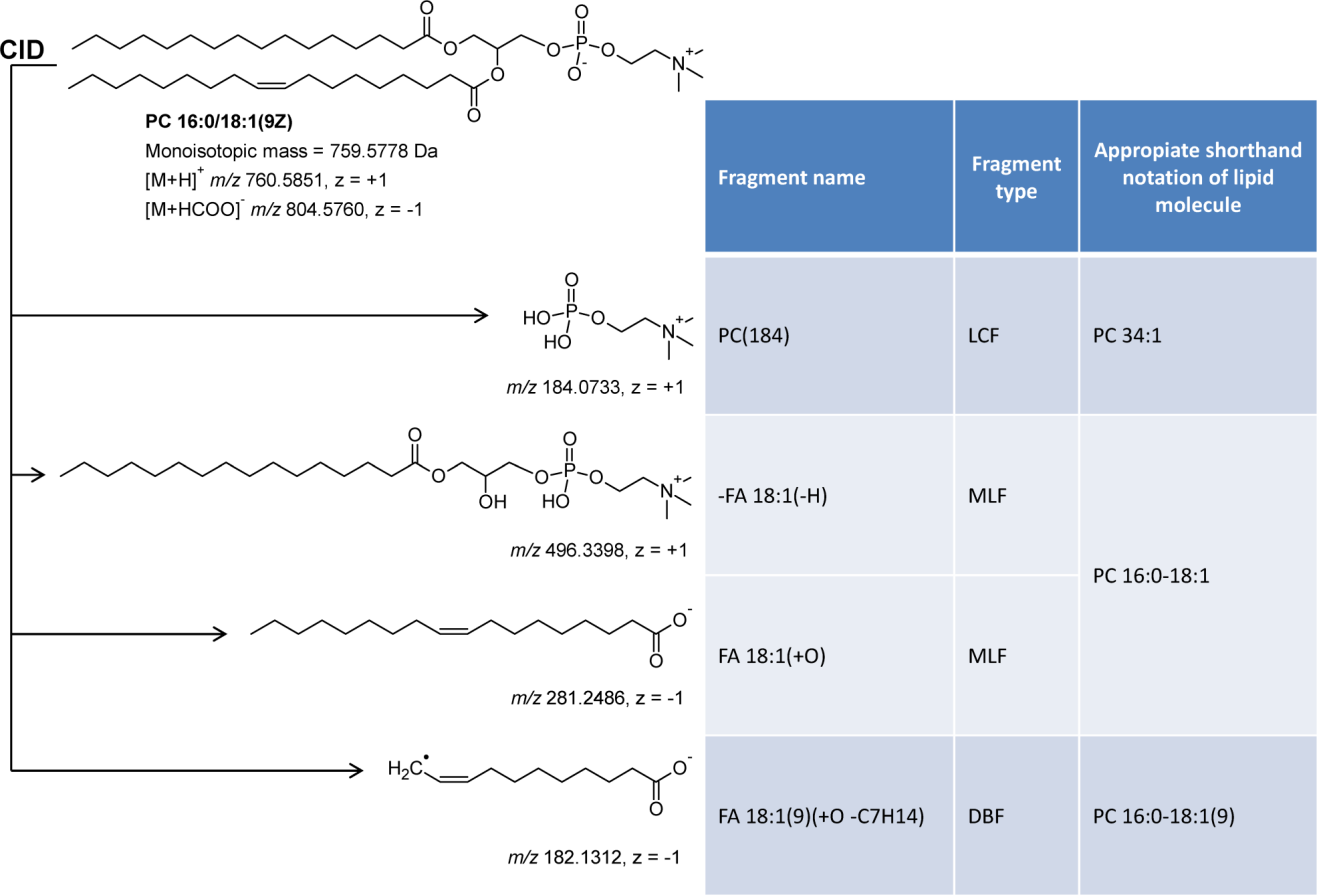
CID of lipid molecules produces several types of fragments that can be used for annotating intact lipid molecules at three different levels. The shorthand notation of the fragment ions is described in the sections: Results and discussion, and S1 Text. LCFs, lipid class-selective fragments; MLFs, molecular lipid species-specific fragments; DBFs, double bond location-specific fragments.

Lipid fragments can be classified into several types depending on their structural attributes and relationship to the structure of the intact lipid precursor molecule (**Fig 1**). This classification has yet to be implemented into a cheminformatics framework (as we do herein), but exists today on a more unintuitive practical level that is implicit in the guidelines for appropriate shorthand notation of intact lipid molecules [20]. As such, one type of lipid fragments is defined as *lipid class-selective fragments* (LCFs) that are characterized by the property that all lipid molecules belonging to the same lipid class yield the same fragment. Examples of LCFs include the fragment ion *m/z* 184.0733 released from all protonated LPC, PC, PC O- and SM molecules and neutral loss 141.0191 released from protonated and sodiated PE and ether PE (PE O-) molecules. Based on the guidelines for shorthand notation, lipid molecules detected by LCFs (and their intact *m/z*) must be annotated at the “lipid species level”, where information on the lipid class followed by the total number of carbon atoms, double bonds and hydroxyl groups present in all hydrocarbon chains are denoted (e.g. PC 34:1, SM 34:1;2, PE O-40:7, TAG 54:3, SE 45:3; see section “Annotation of Lipid Molecules” under Materials and Methods for detailed description of nomenclature used for shorthand notation of intact lipids). Another fragment type is *molecular lipid species-specific fragments* (MLFs) that provide information about the chemical composition of the hydrocarbon chain of individual lipid molecules, such as FAs, alkanols and alkenols (i.e. plasmanyl or plasmenyl chains, respectively), long chain bases (LCBs) and sterols. Based on the guidelines for shorthand notation, lipid molecules detected by MLSs can be annotated at the “molecular lipid species level”, where the lipid class, the number of carbon atoms, C-C double bonds and hydroxyl groups in each of the hydrocarbon chain are denoted (e.g. PC 16:0–18:1, SM 18:1;2/16:0, PE O-18:1p/22:6, TAG 18:1–18:1–18:1, SE 27:1/18:2). Notably, detection of MLFs do not support *de facto* inference of *sn*-1/*sn*-2/*sn*-3-positions of FA moieties in glycero(phospho)lipids. Inferring this information requires validated assays based either on monitoring ratios between MLFs released from positional isomers [29,38] or separating these by LC-MS or ion mobility-MS [39–41]. When using validated assays lipids can be annotated at “hydrocarbon chain position-defined molecular lipid species level”, where the *sn*-positions of the hydrocarbon chains attached to the glycerol-backbone of glycero(phospho)lipids can be denoted (e.g. PC 16:0/18:1, PC 18:1/16:0). A third fragment type is “*double bond location-specific fragments*” (DBFs). Detection of such fragments, for example by using ozone-induced dissociation [42,43], allows annotation of intact lipid molecules at the “double bond location-defined molecular lipid species level”, where the position of double bonds in the hydrocarbon chains are denoted (e.g. PC 16:0–18:1(9)). Notably, DBFs do not allow deciphering whether the orientation of double bonds are *cis* (Z) or *trans* (E). Importantly, the above-described interdependencies between appropriate shorthand notation of intact lipid molecules and structural characteristics of lipid fragments posits that a common nomenclature for annotation of lipid fragments should be able to comply with the guidelines for shorthand notation of intact lipid molecules while at the same time being able to communicate the structural relationship between fragments and the intact lipid precursor molecule.

Here we propose a common nomenclature for annotation of lipid fragment ions. This nomenclature is designed to provide an intuitive and consistent way of pinpointing structural characteristics of fragment ions and reconstructing intact lipid molecules from these without drawing chemical structures or using extensive text description. Furthermore, the nomenclature was constructed to facilitate curation of databases with lipid fragmentation information and downstream computerized data analyses by providing “unique” fragment names for each lipid class as well as a link between the fragments and the precursor molecule. This is achieved by reducing the fragmentation information to minimal structural attributes of lipid molecules that signify either the lipid class or variable hydrocarbon chain features. To demonstrate the utility of the nomenclature we exemplify the annotation of lipid fragment ions derived from representative molecules belonging to five main lipid categories, detected by both shotgun lipidomics and LC-MS^2^-based routines. Moreover, we also present a freely available web application, termed ALEX^123^ lipid calculator, which can be used to access curated lipid fragmentation information for more than 430,000 potential lipid molecules. Finally, we also show that the nomenclature and the ALEX^123^ lipid calculator are applicable for annotating fragments derived from stable isotope-labeled lipid molecules and thereby support high confidence lipid identification in functional studies of lipid metabolic flux.

## Materials and methods

### Chemicals and lipid standards

Chemicals, solvents, and synthetic lipid standards were purchased from Sigma-Aldrich (St. Louis, MO, USA), Rathburn Chemicals (Walkerburn, Scotland) and Avanti Polar Lipids (Alabaster, AL, USA). ^2^H_6_-inositol was from CDN isotopes (Essex, UK), and ^2^H_13_-choline bromide and ^13^C_3_^15^N-serine were from Cambridge isotope laboratories (Cambridge, MA, USA). Yeast extract and peptone were from BD (Lyngby, Denmark). Lipid extract of bovine liver was purchased from Avanti Polar Lipids.

### Yeast cell culture, metabolic labeling and lipid extraction

Exponentially growing *S*. *cerevisiae* (strain BY4742, obtained from EUROSCARF) was cultured at 30°C for 4 hr in YPD medium (1% w/v yeast extract, 2% w/v peptone, 2% w/v glucose) containing 55 μM ^2^H_6_-inositol, 55 μM ^2^H_13_-choline bromide and 300 μM ^13^C_3_^15^N-serine. Cells were killed by adding perchloric acid to a final concentration of 100 mM. Yeast cells were harvested in Eppendorf tubes, washed with ice-cold 155 mM ammonium acetate, frozen in liquid nitrogen and stored at -80°C. Yeast cell lysis and lipid extraction were carried out at 4°C as previously described [44].

### Mass spectrometric lipid analysis

Lipid extracts and synthetic lipid standards were dissolved in chloroform/methanol (1:2, v/v) and subjected to mass spectrometric analysis using an Orbitrap Fusion Tribrid (Thermo Fisher Scientific) equipped with a TriVersa NanoMate (Advion Biosciences), as previously described [1]. In short, aliquots of lipid extracts or synthetic lipid standards were loaded in 96-well plates, mixed with 13.3 mM or 1.3 mM ammonium acetate in 2-propanol for positive and negative ion mode analysis, respectively. Samples were infused using a back pressure of 1.25 psi and ionization voltage of ±0.95 kV. FTMS data were recorded using a max injection time of 100 ms, automated gain control at 2e5, 2 microscans and a target resolution of 500,000 (FWHM at *m/z* 200). FTMS^2^ and FTMS^3^ data were acquired using quadrupole-based CID and ion trap-based resonance-excitation CID with maximum injection time of 100 ms, automated gain control at 5e4, 1 microscan and a target resolution of 30,000. ITMS^3^ data were acquired using max injection time of 200 ms, automated gain control at 1e4 and 1 microscan. All FTMS and ITMS data were acquired using an ion transfer tube temperature of 275°C. MS^ALL^ analysis of mouse plasma, mouse hippocampus and bovine liver was performed as previously described [1,2]. Identification and quantification of lipid molecules detected by FTMS was done using ALEX software [1,2,45].

### Annotation of lipid molecules

Lipid species are annotated as previously described [44,46,47]. At the “lipid species level”, glycero(phospho)lipids are denoted as: <lipid class> <total number of C in hydrocarbon (acyl/alkyl) moieties>:<total number of double bonds in hydrocarbon (acyl/alkyl) moieties> (e.g. PI 34:1). At the “lipid species level”, sphingolipid species are denoted as <lipid class> <total number of C in the long-chain base and acyl moiety>:<total number of double bonds in the long-chain base and fatty acyl moiety>;<total number of OH groups in the long-chain base and acyl moiety> (e.g. SM 35:1;2) [48]. At the “lipid species level”, steryl esters are denoted as <lipid class> <total number of C in the sterol backbone and acyl moiety>:<total number of double bonds in the sterol backbone and acyl moiety> (e.g. SE 45:3).

At the “molecular lipid species level”, glycero(phospho)lipids are denoted as: <lipid class> <number of C in the first hydrocarbon (acyl/alkyl) moiety>:<number of double bonds in the first (acyl/alkyl) moiety>-<number of C in the second acyl moiety>:<number of double bonds in the second acyl moiety> (e.g. PS 16:0–22:6). For triacylglycerols and cardiolipins the third and fourth acyl groups are appended analogously. The acyl groups are indicated in the order of i) increasing carbon number and ii) increasing double bond number. Annotation at the “hydrocarbon chain position-defined molecular lipid species level” is carried out using the expression described above, but replacing the dash (-) separating the acyl moieties by a slash (/). For steryl esters the compositions are denoted as <lipid class> <total number of C in the sterol backbone>:<total number of double bonds in the sterol backbone>/<total number of C in the acyl moiety>:<total number of double bonds in the acyl moiety> (e.g. SE 27:1/18:2).

### ALEX^123^ lipid calculator

ALEX^123^ lipid calculator is a web application available at http://alex123.info/ALEX123/MS.php. It is implemented using PHP and designed for retrieving lipid ionization and fragmentation information stored in the underlying ALEX^123^ lipid database. The ALEX^123^ database is constructed using MySQL and features lipid ionization and fragmentation information (**S1 Table**).

### Automated annotation of fragment ions using lipid data analyzer

Resource LC-MS^2^ data available at MetaboLights (http://www.ebi.ac.uk/metabolights/MTBLS394) were repurposed and processed using Lipid Data Analyzer (LDA) [49] version 2.6.1 [50]. This version of LDA was adapted to support the herein described nomenclature for glycerolipids and glycerophospholipids. For the provided examples we downloaded and used the following two data files: Orbitrap_velos_CID-50_Exp1_014.zip and Orbitrap_velos_CID_pos_50_Exp1_014.zip. These data files feature LC-MS^2^ data on a mixture of 78 synthetic lipid standards analyzed in both positive and negative ion mode using an LTQ Orbitrap Velos Pro (Thermo Scientific) coupled to an reversed-phase LC system [51].

## Results and discussion

### A three-step procedure for shorthand notation of lipid fragment ions

To establish a nomenclature for shorthand notation of lipid fragment ions we first undertook a study to identify commonalities in the fragmentation pathways of lipid molecules. To this end, we performed a comprehensive analysis of lipid fragmentation using structurally-defined lipid molecules from 47 different lipid classes, covering five lipid categories that are common to eukaryotic organisms (**S1 Table**). Using an Orbitrap Fusion mass spectrometer, these lipid molecules were fragmented in both negative and positive ion mode (except for TAG and sterol lipids) using high resolution MS^2^ and MS^3^ analysis with quadrupole-based CID and ion trap-based resonance-excitation CID [1]. As such, the recorded lipid fragmentation data is comparable to that of a broad range of instruments spanning low resolution triple quadrupole and ion trap machines to high resolution hybrid quadrupole time-of-flight, ion trap- and quadrupole-Orbitrap mass spectrometers.

**Fig 2:**
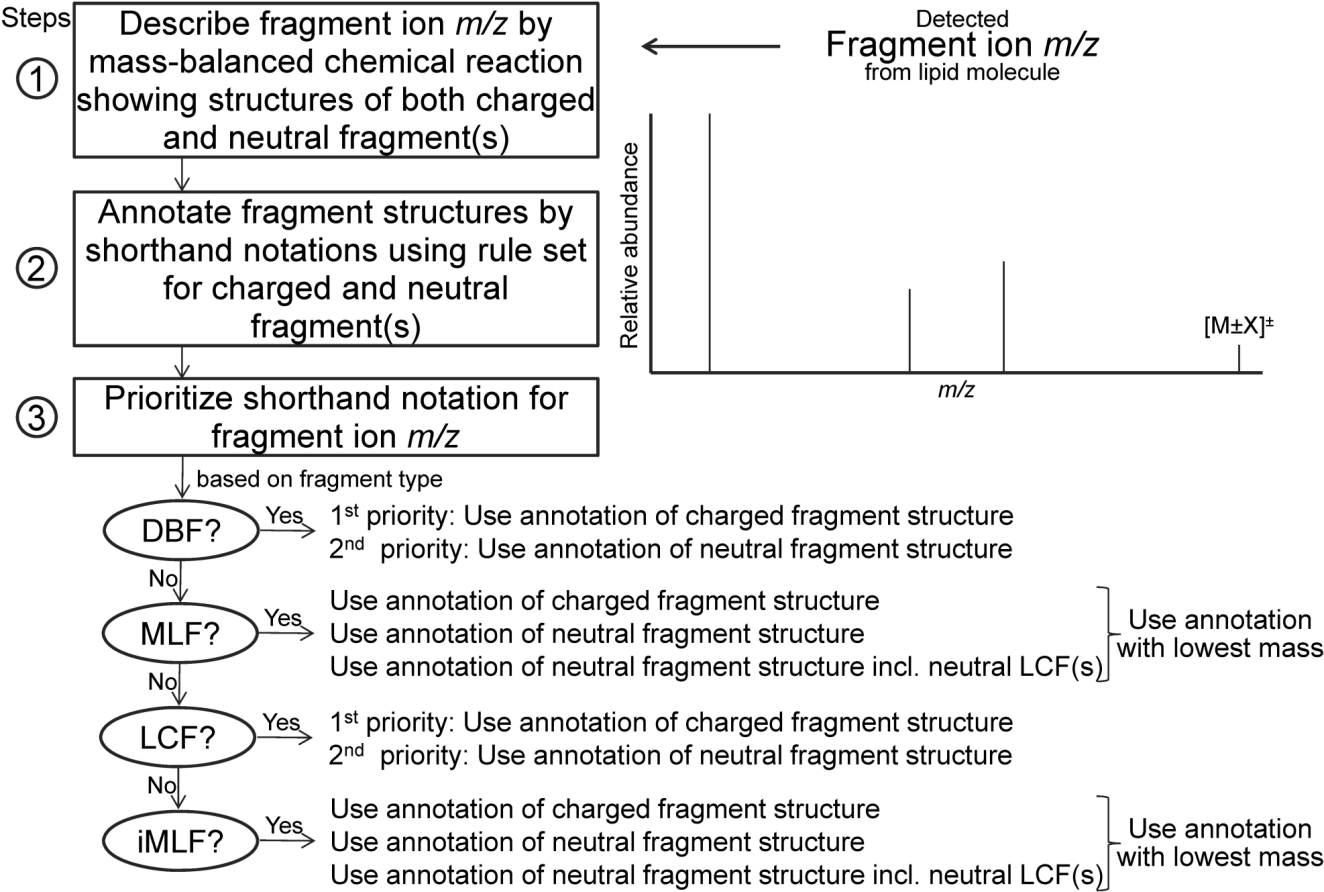
Outline of three-step procedure for implementing shorthand notation of lipid fragment *m/z* values. Step 1: Detected fragment ion *m/z* values are first recapitulated using mass-balanced chemical reactions showing putative structures of both charged and neutral fragments. Step 2: These fragments are then annotated using fragment type-specific annotation rules (described in detail in **S1 Text**). Step 3: Prioritizing the nomenclature to use for shorthand notation of detected fragment ion *m/z* values is based on fragment type, charge and mass difference between charged fragments and composites of neutral fragments (also described in detail in **S1 Text**). Note that the shorthand notation of fragment ion *m/z* values can be based on combinations of fragment types (i.e. DBFs, MLFs, LCFs and iMLFs).

**Fig 3:**
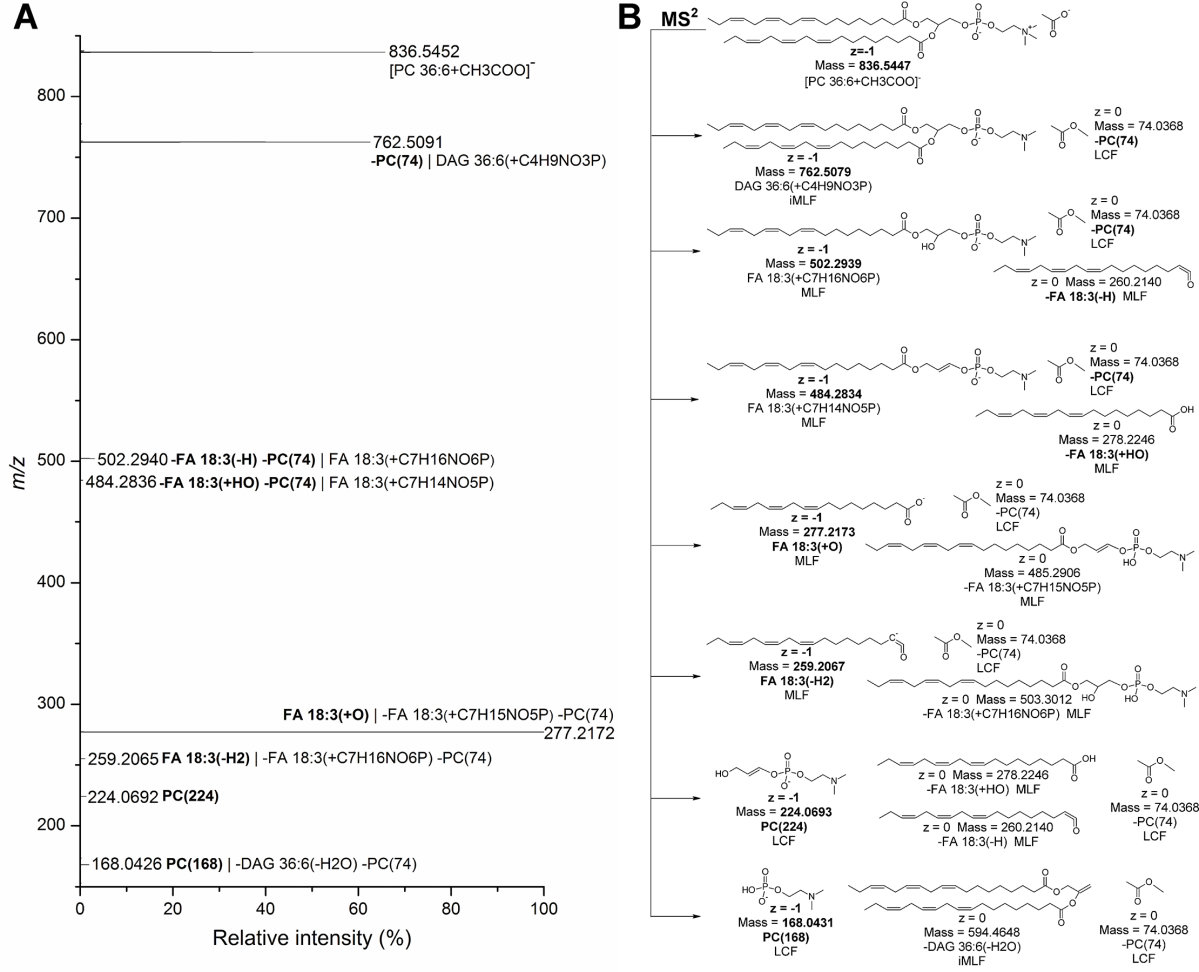
Use mass-balanced chemical reactions to recapitulate lipid fragmentation. A) Negative FTMS^2^ spectrum of *m/z* 836.5, corresponding to the acetate adduct of PC 18:3–18:3. The precursor ion is annotated at the lipid species level (i.e. PC 36:6) since that the composition of FA moieties cannot be inferred from the *m/z* value. Prioritized shorthand notation of fragment *m/z* values are in boldface and implemented according to the three-step procedure shown in **Fig 2**. Non-prioritized (redundant) shorthand notation is shown non-boldface and separated from the prioritized shorthand notation by “|”. B) Overview of fragmentation pathways for [PC 18:3–18:3+CH3COO]^-^ with putative structures of neutral and charged fragments. Note that each chemical reaction is mass-balanced (i.e. the total mass of all fragments equal the mass of the intact precursor molecule). Each structure is represented with charge, monoisotopic mass, shorthand notation and fragment type. Note that neutral (shown on the right) and charged (shown on the left) fragments are prefixed with and without a minus sign “-“, respectively. The annotations shown in boldface (prioritized) are based on annotation rules outlined in **Fig 2** (step 3). LCF, lipid class-selective fragment; MLF, molecular lipid species-specific fragment, iMLF, intermediate MLF.

**Fig 4:**
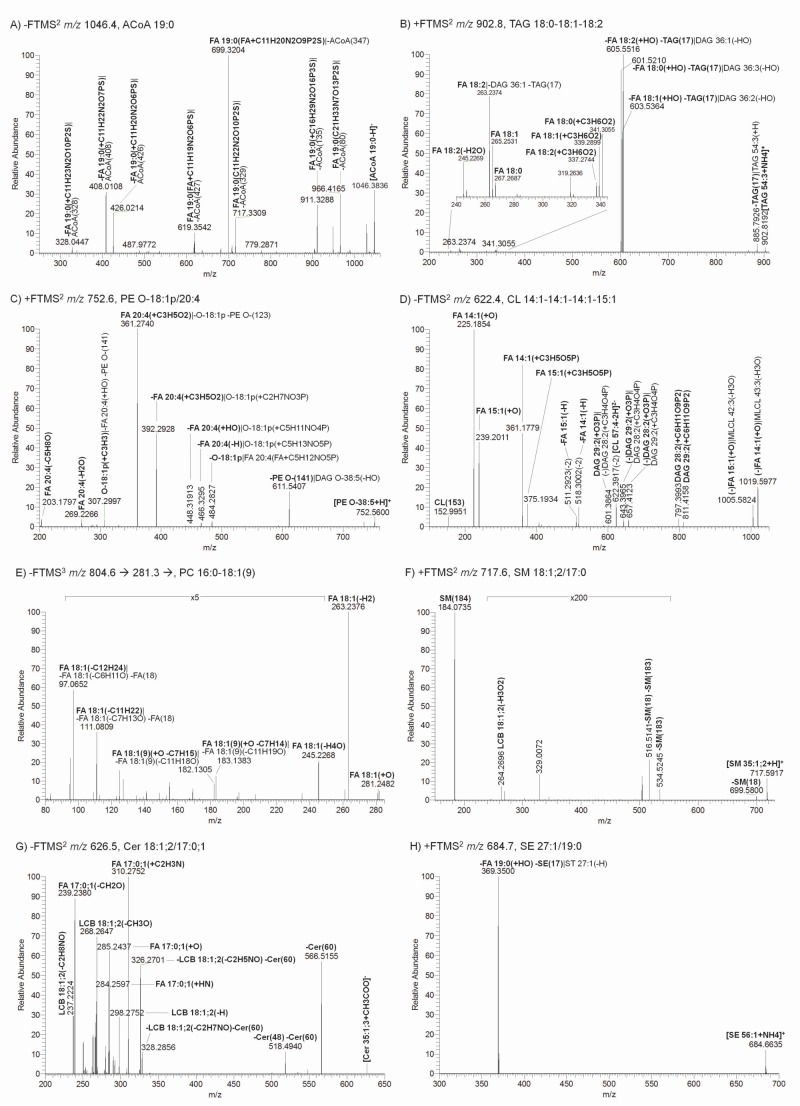
Annotated fragment ion spectra of representative lipid molecules from five different lipid categories. Fragment ion *m/z* values are denoted according to the three-step procedure outlined in **Fig 2**. The shorthand notation includes nomenclature based on both charged and neutral fragments (separated by “|”) (step 2). Annotation shown in boldface is prioritized based on the guidelines outlined in **Fig 2** (step 3). Non-prioritized shorthand notation is occasionally omitted to avoid overly congested mass spectra. The representation of fragment ion *m/z* values by mass-balanced chemical reactions and fragment structures are shown in **S3 Fig** (step 1). A) Negative FTMS^2^ spectrum of deprotonated ACoA 19:0. B) Positive FTMS^2^ spectrum of ammoniated TAG 18:0–18:1–18:2. C) Positive FTMS^2^ spectrum of protonated PE O-18:1p/20:4. D) Negative FTMS^2^ spectrum of deprotonated and doubly charged CL 14:1–14:1–14:-15:1. E) Negative FTMS^3^ spectrum of FA 18:1 carboxylate anion *m/z* 281.3 derived from PC 16:0–18:1(9). F) Positive FTMS^2^ spectrum of protonated SM 18:1;2/17:0. G) Negative FTMS^2^ spectrum of deprotonated Cer 18:1;2/17:0;1. H) Positive FTMS^2^ spectrum of ammoniated SE 27:1/19:0 (cholesteryl ester 19:0).

To systematically annotate detected fragment ions across the five categories of lipids and the different analytical conditions we devised a procedure featuring three consecutive steps (**Fig 2**). This three-step procedure 1) generalizes lipid fragmentation using mass-balanced chemical reactions showing putative structures of both charged and neutral fragments, 2) annotates both charged and neutral fragments using a generic rule set, and 3) prioritizes to denote detected lipid fragment ions (*m/z* values) using either the shorthand notation of charged fragments or that of neutral fragments. The rationales for each of these steps and guideline for their implementation are described in full detail in **S1 Text** and summarized in the following sections. Fragment ion spectra with shorthand notation for representative lipid molecules spanning five different lipid categories are shown in **Figs 3** and **4**.

### Step 1: Recapitulate lipid fragmentation using mass-balanced chemical reactions

The first step in the procedure entails representation of all detected fragment ion *m/z* values with a series of mass-balanced chemical reactions that for each detected *m/z* value shows structures of the charged fragment and also the corresponding neutral fragment(s) (**Fig 3B** and **S3 Fig**). Generalizing lipid fragmentation in this manner highlights three fundamental concepts that are inherent to our nomenclature rules. First, it becomes evident that any fragment ion *m/z* value can be described in reference to both a charged fragment structure and also to the composite of neutral fragment structures. This is exemplified in **Fig 3** showing, for example, that the PC 18:3–18:3-derived fragment ion with *m/z* 502.2940 can be explained by combined neutral losses of methyl acetate and an FA 18:3 moiety as a ketene and also as a charged fragment having a FA 18:3 moiety linked to a glycerylphosphoryl-N,N-dimethylethanolamine residue. Second, inspecting the structural attributes of lipid fragment structures shows that four types of fragments can be produced by CID: LCFs (lipid class-selective fragments), MLFs (molecular lipid species-specific fragments), DBFs (double bond location-specific fragments) and intermediate molecular lipid species-selective fragments (iMLFs). In brief, LCFs encompass common structures that are released from all lipid molecules belonging to the same lipid class, they have identical mass, and they do not contain a hydrocarbon chain. MLFs are characterized by structures having only one hydrocarbon chain with variations in the number of carbon atoms, double bonds and hydroxyl groups. Depending on the lipid class, these hydrocarbon chains can be classified as FA, alkanol and alkenol (i.e. plasmanyl and plasmenyl groups, respectively), LCB and sterol moieties. iMLFs are characterized by structures having two or more hydrocarbon chains (e.g. *m/z* 762.5091 in **Fig 3B** showing a charged fragment composed of a DAG 36:6 moiety linked to a phosphorylethanolamine-N,N-dimethyl residue). DBFs are characterized by specific cleavage of a C-C double bond (**Fig 4E** and **S3E Fig**). The third concept that becomes evident is that the three fragment types, MLFs, iMLFs and DBFs, can all be described in reference to what we term “*minimal hydrocarbon chain-based attributes*” (HCAs) (**S1 Fig**). Of note, HCAs represent the variable hydrocarbon-based building block of intact lipid molecules, they can be grouped into different classes, and annotation of their structural attributes provides a mean to devise a consistent fragment nomenclature that makes it more intuitive to correlate fragment ion *m/z* values back to the structures of intact lipid molecules.

### Step 2: Use fragment type-specific rules to denote both charged and neutral fragments

The second step in the procedure implements specific rules for shorthand notation of both charged and neutral fragment structures (i.e. not the fragment ion *m/z* value itself). To this end, we have established a generic rule sets for annotating structures of LCFs, MLFs, iMLFs and DBFs. These rules are listed in full detail in the **S1 Text**, and exemplified in **Figs 3 and 4** and **S3 Fig** showing MS^n^ spectra and mass-balanced chemical reactions of representative molecules from five different lipid categories. First, a fundamental rule is that uncharged fragment structures should always be prefixed with a minus sign “-”to indicate neutral loss and charged fragment structures should be denoted without a minus sign. Second, structures of LCFs should be annotated by the lipid class abbreviation and its nominal mass in parentheses (e.g. -PE O-(141), *m/z* 611.5407, signifying the neutral loss phosphoethanolamine from an ether PE, **Fig 4C**). Third, MLFs should be denoted by the class of HCA, its original number of carbon atoms, double bonds and potential hydroxyl groups, and followed by in parentheses specification of any chemical modification listed in accordance to Hill notation [52] (e.g. FA 19:0(+C11H20N2O9P2S), *m/z* 699.3204, signifies a charged fragment structure containing a FA 19:0 moiety linked to a chemical residue derived from the intact ACoA precursor, **Fig 4A**). Fourth, iMLFs should be denoted by the class of HCA, its original number of carbon atoms, double bonds and potential hydroxyl groups, and followed by in parentheses any chemical modifications (e.g. DAG 28:2(+C6H11O9P2), *m/z* 797.3993, signifies a charged fragment structure containing a DAG 28:2 moiety linked to a chemical residue derived from the intact CL precursor, **Fig 4D**). Fifth, DBFs should be denoted by the class of HCA, its original number of carbon atoms, number of double bonds and locations of double bonds, followed by in parentheses any chemical modification (e.g. FA 18:1(9)(+O -C7H15), *m/z* 182.1305, signifying a charged (radical) ion derived from an FA 18:1(9) moiety, **Fig 4E**). Notably, by using the framework of mass-balanced chemical reactions and annotating structures of both neutral and charged fragment structures it becomes evident that a particular fragment ion *m/z* value can be described with dual nomenclature corresponding to both the charged fragment and the composite of all neutral fragments. This possibility for dual shorthand notation of fragment *m/z* values can lead to “congested” mass spectra overfilled with text-based shorthand notations which make it difficult to appreciate the spectral profile. Hence, for spectral annotation it is advisable to prioritize the use of nomenclature based on fragment type and whether the fragment structure(s) is charged or neutral.

### Step 3: Prioritize the shorthand notation to use for spectral annotation

The third and final step in the procedure prioritizes the nomenclature to use for shorthand notation of lipid fragment ion *m/z* values. To this end, we implemented a decision tree-based routine where shorthand notation is prioritized according to fragment type in the following order: DBFs, MLFs, LCFs and iMLFs (**Fig 2**). For example, a fragment ion *m/z* value with specific information on double bond location should be annotated with nomenclature according to DBFs instead of, for example, nomenclature based on composites of other fragment types. Similarly, a fragment ion *m/z* value featuring MLF information, for example, “-FA 18:1(+HO) -TAG(17)” from intact TAG 54:3 should be prioritized over the iMLF “DAG 36:2(-HO)” (**Fig 4B** and **S3B Fig**). A fragment ion *m/z* value featuring LCF information, for example, “SM(184)” from intact SM 35:1;2 should be prioritized over the iMLF “-Cer 35:1;2(-H2O)” (**Fig 4F** and **S3F Fig**). We note that some fragment ion *m/z* values, derived for example from CL molecules, corresponds to the release of two iMLFs and no other fragment types. Hence, these fragment ion *m/z* values should be annotated with shorthand notation based only on iMLFs. For MLFs and iMLFs, the decision tree-based routine also prioritizes whether to use shorthand notation based on nomenclature for charged structures or the composite of neutral losses. In cases where CID yields both a charged MLF and a combination of neutral MLF and a neutral LCF the decision tree-based procedure will prioritize to use the nomenclature according to the fragment structure(s) having the lowest mass (not *m/z*). This scenario is exemplified by the PC 18:3–18:3-derived fragment ion *m/z* 502.2940 that can be annotated as a charged MLF “FA 18:3(+C7H16NO6P)” with mass 502.3 Da and as a neutral composite of an MLF and an LCF “-FA 18:3(-H) -PC(74)” with a total mass of 334.3 Da (**Fig 3B**). According to the decision tree-based routine the fragment ion *m/z* 502.2940 should be annotated as the composite of neutral losses (i.e. “-FA 18:3(-H) -PC(74)”), as this has the lowest mass.

Taken together, the devised three-step nomenclature procedure establishes, for the first time, a generic framework for systematic annotation of detected fragment ion *m/z* values in CID-based MS^2^ and MS^3^ spectra of lipid molecules. Moreover, this framework also provides an avenue for automatically and consistently curating lipid fragmentation information in databases and harnessing the information to support high confidence lipid identification. We note that the framework has been devised to facilitate the matching of fragment ion *m/z* values to structures of LCFs, MLFs, iMLFs and DBFs *that are released from a given precursor lipid upon fragmentation*. This strategy is different to that of other, non-formalized annotations where lipid fragment ion *m/z* values are typically denoted as ‘intact’ lipid molecules using shorthand notation such as “LPA(20:4)-H3O” [53] or using chemical formulas such as “fragment C3H6O5P” [54] or alphabetical symbols (e.g. Y_0_’) [55]; all of which make it difficult to relate the structure of a fragment ion back to the structure of the intact lipid molecule (**S2 Fig**). Furthermore, comparing our nomenclature to that of LipidBlast [54] shows that this software uses only the positional descriptors sn-1, sn-2 and sn-3 to denote MLFs (e.g., “[M‐H‐87]‐sn2+H2O”) and as such does not specify the number of C atoms and double bonds in FA-containing fragments (**S2 Fig**). This might be considered adequate for analysis of synthetic lipid standards where the name and the structure of the lipid molecule are known. However, this nomenclature format will produce misleading spectral annotations and false-positive lipid identifications when used for analyzing complex biological samples where both positional- and structural lipid isomers are present [29,38,41].

### ALEX^123^ lipid calculator

**Fig 5:**
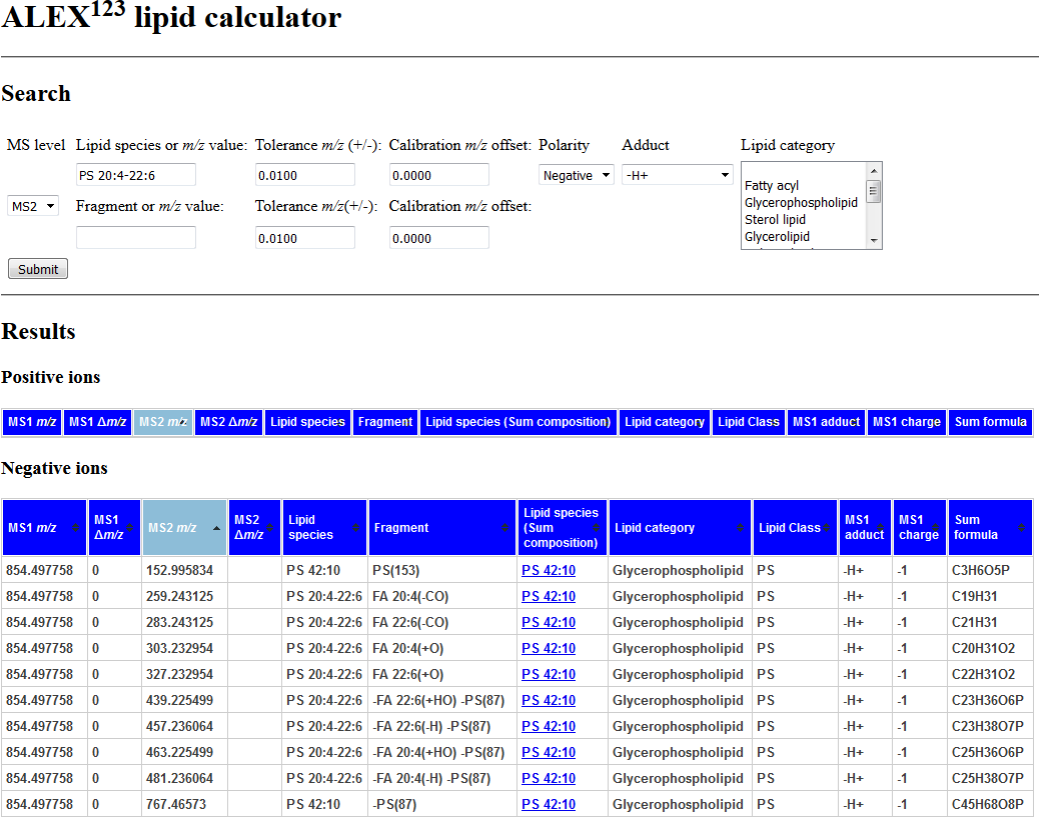
Screenshot of the ALEX^123^ lipid calculator showing MS^2^ information for the molecular lipid species PS 20:4–22:6 (MS^2^ spectrum is shown in Fig 6). The application is freely available at www.alex123.info/ALEX123/MS.php.

Having established a generic framework for annotating lipid fragment ion *m/z* values we next developed a web-based application, termed ALEX^123^ lipid calculator (**Fig 5**), which assists annotation of lipid fragment ions and also helps identify intact lipid molecules with high confidence. Currently, the ALEX^123^ lipid calculator provides ionization information for over 25,000 lipid species from more than 89 lipid classes at the MS^1^ level. Furthermore, the database also features curated MS^2^ and MS^3^ fragmentation information for more than 430,000 molecular lipid species covering 49 different lipid classes (**S1 Table**). To our knowledge, this is currently the most comprehensive freely available resource with curated information on lipid ionization and fragmentation. At the level of MS^1^ analysis, lipid molecules are annotated at the “lipid species level”. At the level of MS^2^ and MS^3^ analysis, lipids are annotated at the “molecular lipid species level” when represented by MLFs and the “lipid species level” when represented by LCFs or iMLFs. Available MS^2^ information includes *m/z* values of fragment ions and corresponding shorthand notations based on the above-described three-step procedure (**Fig 2**). We note that the spectral information in the ALEX^123^ lipid calculator also features additional metadata that for all lipid molecules and fragment ions specifies lipid category, lipid class, adduct ion, charge and chemical formula. To support structural elucidation, the ALEX^123^ lipid calculator has also been equipped with various search fields that allow users to simultaneously specify names of lipid molecules, measured *m/z* values of intact lipid precursor ions and fragment ions, adduction, polarity and *m/z* tolerances.

### Spectral annotation facilitates high confidence lipid identification

To exemplify how the nomenclature for shorthand notation of lipid fragment ions facilitates confident lipid identification we manually shortlisted a set of low abundance lipid molecules for which fragment ion intensity is expected to be of poorer quality as compared to fragment ions derived from more abundant lipid molecules. These lipid molecules were detected by MS^ALL^ analysis of mouse plasma [2], mouse hippocampus [1] and bovine liver.

First, high resolution FTMS^1^ analysis of mouse plasma detected a low abundance signal at *m/z* 424.3413, corresponding to protonated acyl carnitine (ACar) 18:2 (-2 ppm mass accuracy) (**S4A Fig**). FTMS^2^ analysis of *m/z* 424.3 detected two fragment ions listed in the ALEX^123^ database that match fragment ions expected to be derived from protonated ACar 18:2 (**S4B Fig**). The fragment ion with *m/z* 263.2362 matches the MLF FA 18:2 (-2.8 ppm mass accuracy) and the ion at *m/z* 365.2667 matches the MLF FA 18:2(+C4H6O3) (-5.3 ppm mass accuracy). Detection of these structure-specific fragment ions, and the intact lipid molecule by FTMS^1^, demonstrated specific detection of ACar 18:2 in mouse plasma.

**Fig 6:**
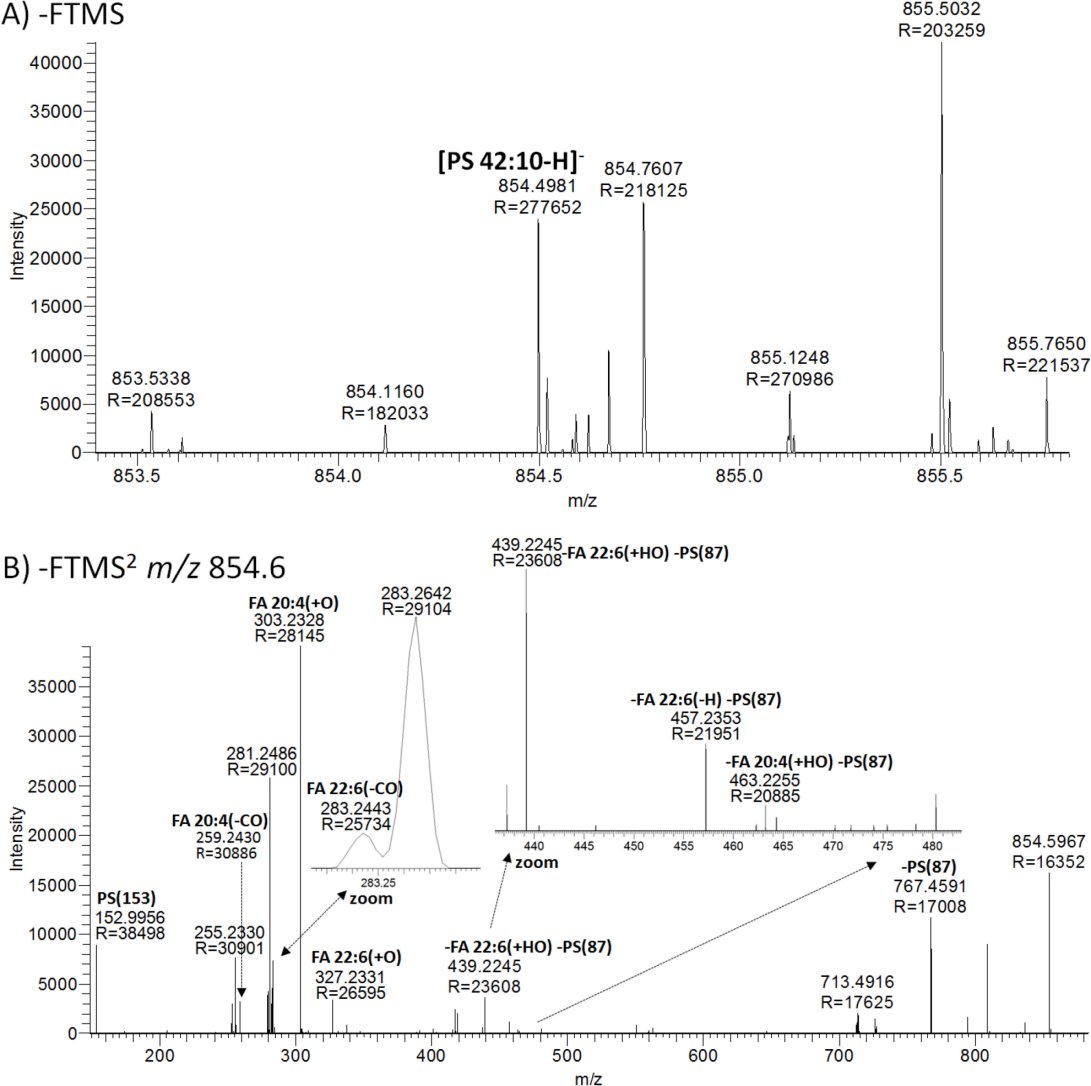
Identification of PS 20:4–22:6 in mouse hippocampus. A) Negative FTMS spectrum of mouse hippocampus. The precursor ion matching deprotonated PS 42:10 is highlighted in boldface. B) Negative FTMS^2^ spectrum of *m/z* 854.6 with detection of MLFs and LCFs matching PS 20:4–22:6, annotated in boldface.

Second, high resolution FTMS^1^ analysis of mouse hippocampus detected a low abundance signal at *m/z* 854.4981, matching deprotonated PS 42:10 (0.4 ppm mass accuracy) (**Fig 6A**). At first hand, this highly unsaturated PS molecule was somewhat puzzling and difficult to reconcile with lipid metabolic pathways in mammalian cells. FTMS^2^ analysis of *m/z* 854.6 detected two PS-derived LCFs at *m/z* 152.9956 and *m/z* 767.4591 annotated as “PS(153)” and “-PS(87)”, respectively (**Fig 6B**). This spectral information confidently identifies the molecule as PS 42:10 at the “lipid species level”. The FTMS^2^ analysis also detected seven out of eight possible MLFs listed in the ALEX^123^ database for PS 20:4–22:6 (**Fig 5**) with a mass accuracy better than 4.1 ppm. These MLFs include the FA carboxylate anions FA 20:4(+O) and FA 22:6(+O), their decarboxylated counterparts (e.g. FA 22:6(-CO)) and fragments corresponding to the neutral loss of the FA moieties (e.g. -FA 20:4(+HO) -PS(87)). This information univocally demonstrates that the mouse hippocampus lipidome includes the highly polyunsaturated and low abundance molecular glycerophospholipid species PS 20:4–22:6 (corresponding to 0.15% of all PS molecules, data not shown). Of note, our data is corroborated by previous report indicating the presence of PS 20:4–22:6 PS in mouse brain [56] and raises the mechanistic questions as to how it is synthesized and what its molecular functions are?

To exemplify the use how the fragment nomenclature supports confident lipid identification using MS^3^ fragmentation we selected a low abundance TAG species detected in bovine liver. High resolution FTMS^1^ analysis detected ammoniated TAG 52:2 at *m/z* 876.8015 (3.8 ppm mass accuracy, **S5A Fig**). FTMS^2^ analysis of *m/z* 876.7 detected a LCF at *m/z* 859.7749, corresponding to neutral loss of ammonia and annotated as “-TAG(17)” (**S5B Fig**). The FTMS^2^ analysis also detected of nine out of twelve possible MLFs matching low abundance TAG 16:0–18:0–18:2 with a mass accuracy better than 4.9 ppm, and seven of eight MLFs matching the much more abundant isomeric TAG 16:0–18:1–18:1 (not discussed in further detail). The TAG 16:0–18:0–18:2-derived MLFs includes the neutral loss-derived fragments “-FA 18:0(+HO) -TAG(17)” at *m/z* 575.5059, “-FA 18:2(+HO) -TAG(17)” at *m/z* 577.5215 and “-FA 16:0(+HO) -TAG(17)” at *m/z* 603.5373. The MLFs also includes three low abundance FA 16:0, FA 18:2 and FA 18:0 acyliums. Moreover, the MLF FA 18:2(-HO) was also detected, but the complementary FA 16:0 and FA 18:0 fragments were not detected. Subjecting the fragment ions with *m/z* 575.5 and *m/z* 603.5 to in-depth ITMS^3^ analysis revealed the above-mentioned FA 16:0, FA 18:2, FA 18:0 acyliums and FA 18:2(-HO) (**S5C Fig** and **S5D Fig**). Taken together, these fragment ions confidently identify the low abundance molecular lipid species TAG 16:0–18:0–18:2 in the background of the much more abundant isomeric species TAG 16:0–18:1–18:1.

### Automated annotation of lipid fragment ions detected by LC-MS^2^ analysis

**Fig 7:**
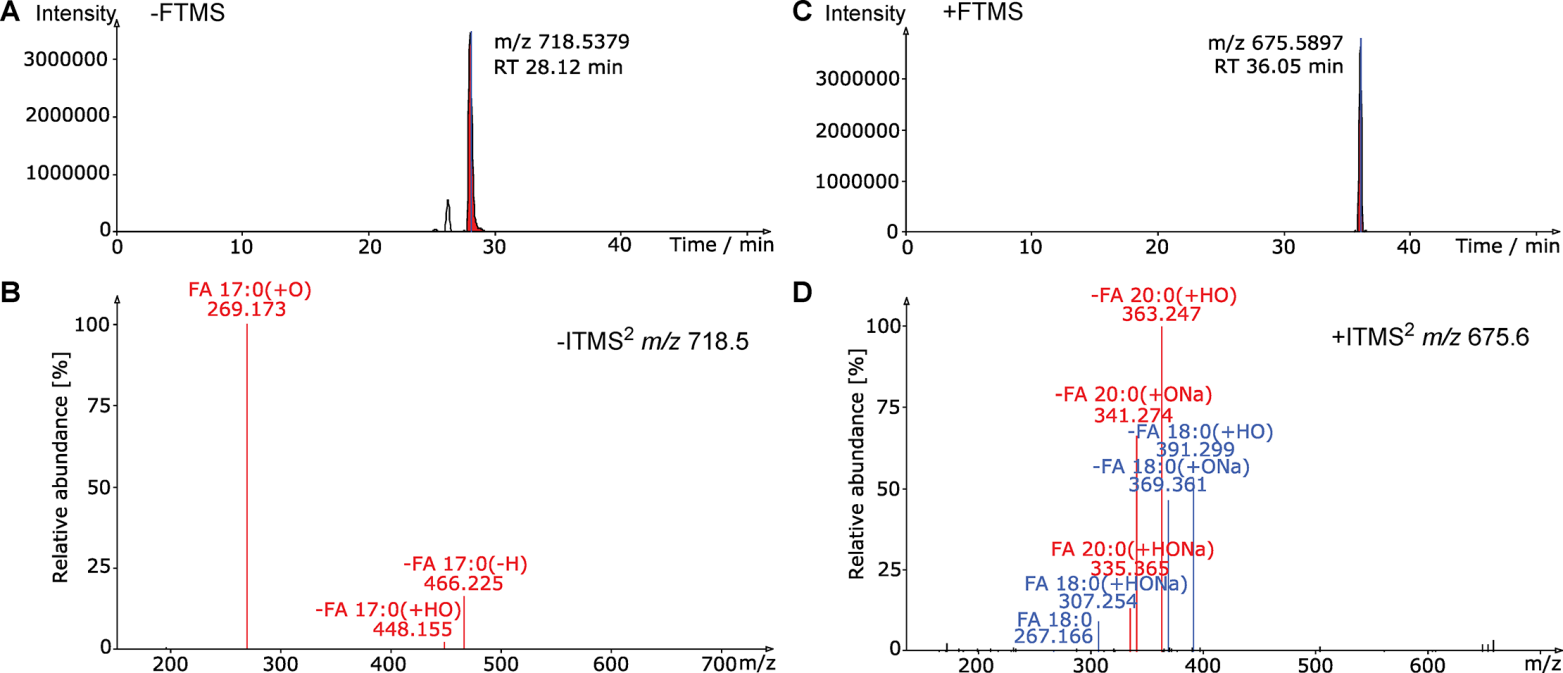
LDA software supports automated annotation of lipid fragment ions. A) Negative ion mode extracted ion chromatogram of *m/z* 718.5379±0.013, corresponding to deprotonated PE 34:0 (i.e., synthetic standard PE 17:0–17:0). B) Negative ion mode FTMS^2^ spectrum of *m/z* 718.5. Fragment ions are automatically annotated by LDA and collectively used to identify the molecular lipid species PE 17:0–17:0. C) Positive ion mode extracted ion chromatogram of *m/z* 675.5897±0.013, corresponding to sodiated DAG 38:0 (i.e., synthetic standard DAG 18:0–20:0). D) Positive ion mode FTMS^2^ spectrum of *m/z* 675.6. Fragment ions are automatically annotated by LDA and collectively used to identify the molecular lipid species DAG 18:0–20:0.

As a proof of concept we subsequently embedded our nomenclature rules in LDA (Lipid Data Analyzer) [49], a software supporting automated high confidence lipid identification and quantification [50]. In addition to using multiple lipid fragment ions to support lipid identification and outputting quantitative information of lipids identified at the molecular lipid species-level this software also features a convenient user-interface for reviewing individual MS^2^ spectra in which fragment ions can be automatically annotated (**Fig 7**). To exemplify the possibility to automatically annotate detected lipid fragment ions we made use of a resource dataset featuring LC-MS^2^ data on a lipid standard mixture containing 78 different synthetic standards, including PE 17:0–17:0 and DAG 18:0–20:0.

Among the signals detected in the negative ion mode LC-MS data was a precursor ion with *m/z* 718.5379 eluting at 28.12 min, which matches deprotonated PE 34:0 **(Fig 7A)**. ITMS^2^ analysis of this precursor ion showed fragment ions at *m/z* 269.2, 448.2 and 466.2 which the LDA automatically annotated as FA 17:0(+O), -FA 17:0(+HO) (fatty acid loss) and -FA 17:0(-H) (ketene loss), respectively (**Fig 7B**). Collectively, these fragment ions unequivocally identify the precursor ion as the molecular lipid species PE 17:0–17:0. In the data acquired from of the same lipid mixture in positive ion mode we shortlisted a precursor ion with *m/z* 675.5897 eluting at 36.0 min, which corresponds to sodiated DAG 38:0 (**Fig 7C**). ITMS^2^ analysis of this molecule yielded fragment ions with *m/z* 391.3, 369.4, 363.3, 341.3, 335.4, 307.3 and 267.2, which LDA automatically annotated as -FA 18:0(+HO), -FA 18:0(+ONa), -FA 20:0(+HO), -FA 20:0(+ONa), FA 20:0(+HONa), FA 18:0(+HONa) and FA 18:0, respectively. (**Fig 7D**). Collectively, these fragment ions unequivocally identify the precursor ion as the molecular lipid species DAG 18:0–20:0. Taken together, these examples demonstrate that the proposed nomenclature system not only facilities manual lipid identification (as outlined in the previous section), but can also be used in conjunction with software-based routines to easily verify the fidelity of automated lipid identifications.

### The nomenclature is applicable for annotation of stable isotope-labeled lipids

To evaluate the generic nature of the nomenclature system we inspected its applicability to the emerging field of “dynamic lipidomics”, which uses metabolic incorporation of stable isotope-labeled precursors and mass spectrometric analysis to monitor lipid metabolic flux [57–59]. Such investigations can be performed by feeding cells or animals with a wide range of metabolic precursors labeled with ^13^C, ^2^H, ^15^N and ^18^O. Depending on the organism, stable isotope-labeled precursors can be incorporated into different structural attributes of a lipid molecule and when labeling with a cocktails of precursors the stable isotope-labeled precursors can be incorporated simultaneously into a single lipid molecule. This yields an additional dimension of lipid structural complexity that can be harnessed using high resolution MS^ALL^ technology [1]. However, the increased lipid structural complexity also calls for implementation of a systematic nomenclature that can adequately denote fragment ions derived from molecular lipid species having distinct structural attributes labeled with stable isotopes.

**Fig 8:**
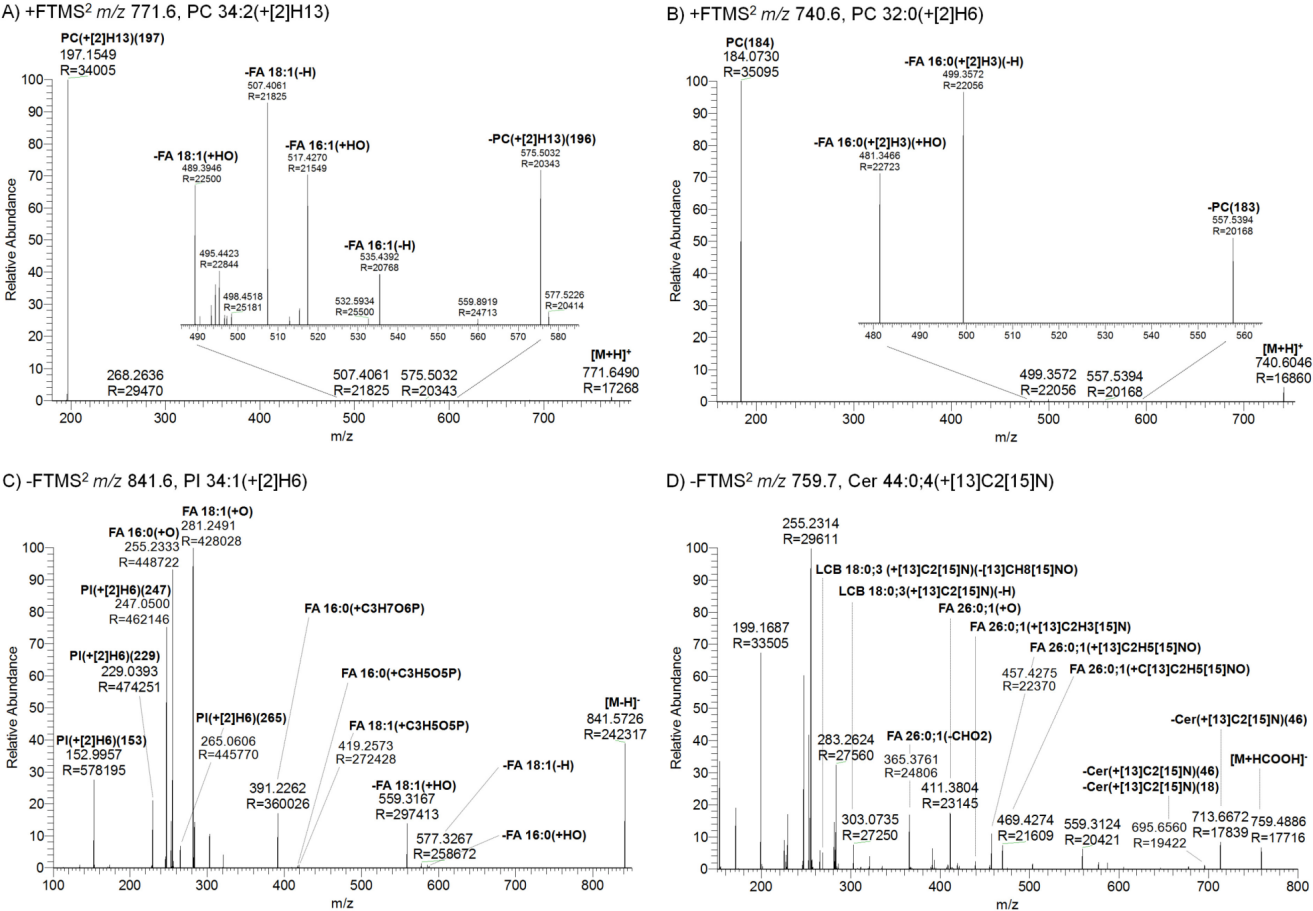
Annotation of fragment ions from stable isotope-labeled lipids. A) Positive FTMS^2^ spectrum of protonated PC 34:2(+[2]H13). The fragment ions identify the molecular lipid species as PC(+[2]H13) 16:1–18:1. B) Positive FTMS^2^ spectrum of protonated PC 32:0(+[2]H6). The fragment ions identify the molecular lipid species as PC 16:0(+[2]H3)-16:0(+[2]H3). C) Negative FTMS^2^ spectrum of deprotonated PI 34:1(+[2]H6). The fragment ions identify the molecular lipid species PI(+[2]H6) 16:0–18:1. D) Negative FTMS^2^ spectrum of the formate adduct of Cer 44:0;4(+[13]C2[15]N). The annotated fragment ions identify the molecular species as Cer 18:0;3(+[13]C2[15]N)/26:0;1. Note that non-annotated fragment ions derive from co-isolated lipids. Fragmentation diagrams for the lipid molecules and indicated fragment ion *m/z* values are shown in **S6 Fig**.

To support shorthand notation of fragment ions derived from stable-isotope labeled lipids we extended the rule set of the fragment nomenclature. Full details of these rules are provided in **S1 Text**. First we extended the guidelines for shorthand notation of intact lipid molecules at the “lipid species level” and the “molecular lipid species level” [20]. To the guidelines for “lipid species” we added that stable isotopes should be specified by using the recommended shorthand notation followed by in parentheses a “+” sign, the heavy nuclei indicated by their isotope number in squared brackets, their atomic symbol and their numbers, listed in accordance to Hill notation (*e*.*g*. PC 34:1(+[2]H9), Cer 44:0;4(+[13]C2[15]N), **Fig 8**). To the guidelines for “molecular lipid species” we added that the naming convention for stable isotopes should follow the structural attributes into which they are incorporated (*e*.*g*. PC(+[2]H13) 16:0–181, PC 16:0(+[2]H3)-16:0(+[2]H3), **Fig 8**).

For annotation of fragment ions we subsequently added the naming convention for stable isotopes into the rule sets for LCFs, MLFs, iMLFs and DBFs. This extension is exemplified in **Fig 7** showing fragment ion spectra from four representative lipid molecules labeled with different configurations of heavy nuclei. Of note, LCFs are denoted by the lipid class abbreviation followed first by specification of stable isotopes and then by the nominal mass in parentheses. For example, “PC(+[2]H13)(197)” and “PI(+[2]H6)(247)” indicate charged phosphocholine and phosphoinositol structures labeled with thirteen ^2^H atoms and six ^2^H atoms, respectively (**Fig 8A and 8C**). Similarly, MLFs are denoted by HCA abbreviation followed first by specification of stable isotopes and then by any chemical modifications in parentheses. For example, the fragment ion with *m/z* 499.3572 in **Fig 8B** is denoted as “-FA 16:0(+[2]H3)(-H)” to indicates neutral loss of an FA 16:0 moiety having three ^2^H atoms as a ketene. Moreover, the fragment ion with *m/z* 268.2344 in **Fig 8D** is denoted as LCB 18:0;3(+[13]C2[15]N)(-[13]CH8[15]NO) to indicate a charged LCB fragment originally having two ^13^C and one ^15^N incorporated but after fragmentation having lost a chemical feature corresponding to one ^13^C, eight H, one ^15^N and one O.

In summary, the ability of the fragment ion nomenclature to consistently account for shorthand notation of also stable-isotope labeled lipid fragment ions demonstrates its generic nature and highlight that it can readily be extended to describe a wide range of fragment structures and accurately match these to structure-specific fragment ions detected by MS^n^ analysis. We note that the ALEX^123^ lipid calculator at the present features 28 lipid classes labeled with stable isotopes that can be generated when feeding cells or animals with the commercially available metabolic precursors ^2^H_9_-choline, ^2^H_3_-methionine, ^2^H_6_-inositol, ^13^C_3_^15^N-serine and their combinations (**S1 Table**).

## Conclusions

In this report we have outlined a generic framework for shorthand notation of lipid fragment ions. The framework consists of a three-step procedure that systematically recapitulates lipid fragmentation using mass-balanced chemical reactions showing charged and neutral fragment structures, uses defined rules for annotating specific types of fragments, and uses a decision tree-based routine for implementing shorthand notation of detected fragment ion *m/z* values in mass spectra. We have demonstrated that the nomenclature is able to systematically and consistently describe structural details of fragment ions released upon CID of unlabeled and stable isotope-labeled molecular lipid species encompassing 47 lipid classes and five different lipid categories. Furthermore, we have shown that the nomenclature can be computerized and made searchable in the online ALEX^123^ lipid calculator to support both manual and automated high confidence lipid identification in biological sample matrices. Notably, the fragment nomenclature framework also provides an avenue to develop new algorithms for automated high confidence lipid identification in high throughput lipidomics studies. As such, the text-based fragment nomenclature and “substrings” thereof can be queried using algebraic string-based operators available in all programming languages (e.g., C++, SAS). This text-based information can, for example, be harnessed for counting the frequency of specific fragment types across large number of samples and also for implementing complementarily filters to secure high confidence lipid identification (e.g., mandatory detection of two FA-based fragments either as matching acyl anions, as matching loss of ketenes or as matching loss of fatty acids). Based on its systematic design and its ability to be easily computerized we deem that our proposed fragment nomenclature can become a valuable addition to the expanding palette of cheminformatics tools that are being developed to assist the characterization of lipid molecules in biological systems. Finally, we note that the nomenclature is applicable to annotation of MS^n^ spectra of lipid molecules acquired by both direct infusion-based (i.e. shotgun) and LC-based lipidomics techniques, and also mass spectrometry imaging approaches.

## Supporting information

S1 Text Detailed description of rules and rationales for annotating lipid fragment ions. (DOCX)

S1 Table List of lipid classes and lipid molecules for which MS^1^, MS^2^ and MS^3^ information is available in the online ALEX^123^ lipid calculator. (XLSX)

S1 Fig Examples of minimal HCAs (hydrocarbon chain attributes) used for annotating neutral and charged fragment structures. (PDF)

S2 Fig Comparison of nomenclatures for shorthand notation of lipid fragment ions. (PDF)

S3 Fig Mass-balanced chemical reactions and annotation of fragment structures related to MS^n^ spectra of lipid molecules shown in Fig 3. (PDF)

S4 Fig Identification of low abundance ACar 18:2 in mouse plasma. (PDF)

S5 Fig Identification of low abundance TAG 16:0–18:0–18:2 in bovine liver. (PDF)

S6 Fig Mass-balanced chemical reactions and annotation of fragment structures related to MS^n^ spectra of stable isotope-labeled lipid molecules shown in Fig 6. (PDF)
