# Supplementary material for: Proposal for a common nomenclature for fragment ions in mass spectra of lipids
Source: PLoS One. 2017 Nov 21;12(11):e0188394. doi: 10.1371/journal.pone.0188394 (PMC5697860; doi:10.1371/journal.pone.0188394)
Supplement: S1 Text — (DOCX) [file pone.0188394.s001.docx]

**S1 Text**

This section outlines the rationales behind the devised nomenclature rules and provides additional examples of its implementation. This supplementary text features the following subsections:

- Recapitulating lipid fragmentation by mass-balanced chemical reactions.

- Definitions of lipid fragment types.

- Definition of minimal hydrocarbon chain-based attributes (HCAs).

- Rules for shorthand notation of different fragment types.

- Rules for shorthand notation of fragment ions in mass spectra.

- Rules for annotating intact stable isotope-labeled lipids and their fragments.

**Recapitulating lipid fragmentation by mass-balanced chemical reactions**

The framework for describing lipid fragmentation by using mass-balanced chemical reactions and tentative structures of both charged fragments and their corresponding neutral fragment(s) is exemplified in **Fig 3** (in main text). This figure shows an annotated FTMS^2^ spectrum of synthetic PC 18:3-18:3 (panel A) together with a set of mass-balanced reactions showing tentative structures of both charged fragments and their corresponding neutral fragment(s) (panel B). Using this framework demonstrates that the fragment ion with *m/z* 762.5091 can be explained by neutral loss of methyl acetate (74.0386 Da) from the intact precursor ion yielding a charged fragment featuring a DAG 36:6 moiety linked to a phosphorylethanolamine-N,N-dimethyl residue. Similarly, the fragment ion with *m/z* 502.2940 can be explained by combined neutral losses of methyl acetate and a FA 18:3 moiety as a ketene yielding a charged fragment having a FA 18:3 moiety linked to a glycerylphosphoryl-N,N-dimethylethanolamine residue. Also the fragment ion with *m/z* 484.2836 can be explained by loss of methyl acetate but with additional neutral loss of a FA 18:3 moiety as an acid producing a charged fragment with an FA 18:3 moiety linked to a dehydrated glycerylphosphoryl-N,N-dimethylethanolamine residue. The prominent fragment ion with *m/z* 277.2172 corresponds to a carboxylate anion of FA 18:3, which can be explained by the combined neutral losses of methyl acetate and an FA 18:3 moiety linked to a dehydrated glycerylphosphoryl-N,N-dimethylethanolamine residue. The low abundance fragment ion with *m/z* 259.2065 corresponds to a dehydrated fragment of the FA 18:3 carboxylate anion, which can derive from neutral loss of H_2_O from the FA 18:3 carboxylate anion and also from charge-mediated neutral loss of a FA 18:3 moiety linked to a glycerylphosphoryl-N,N-dimethylethanolamine. The other low abundance fragment ion with *m/z* 224.0692 corresponds to deprotonated, dehydrated and charged glycerylphosphoryl-N,N-dimethylethanolamine, which can be produced by combined neutral losses of methyl acetate and each of the FA 18:3 moieties as a ketene and an acid. Finally, the fragment ion with *m/z* 168.0426 corresponds to deprotonated and charged ethanolamine-N,N-dimethyl produced by combined neutral losses of methyl acetate and a dehydrated DAG 36:6 moiety. Importantly, by using mass-balanced chemical reactions to describe the fragmentation behavior of PC 18:3-18:3 it becomes evident that all detected fragment ion *m/z* values can be described as both charged fragment structures and as loss of neutral fragment structures. Moreover, it also shows that neutral and charged fragments can be characterized as structural attributes containing hydrocarbon chains (which for PC 18:3-18:3 are either a FA 18:3 moiety or a DAG 36:6 moiety) or lipid class-dependent structural attributes (as exemplified by the loss of methyl acetate and the charged ethanolamine-N,N-dimethyl fragment ion with *m/z* 168.0426).

**Definitions of lipid fragment types**

By generalizing positive and negative ion mode MS^2^ and MS^3^ fragmentation using mass-balanced chemical reactions for lipid molecules spanning 49 different lipid classes (**S1 Table**) we pinpointed a set of highly conserved fragmentation patterns that always produce a combination of four distinct types of fragments. These fragment types can be defined as:

1. LCFs (lipid class-selective fragments) which are characterized by the properties that they can be both neutral and charged, are released from all lipid molecules belonging to the same lipid class and have identical mass, and they do not contain a hydrocarbon chain. Thus, LCFs provide information about structural attributes that are common to all molecules of a particular lipid class (e.g. a head group of a glycerophospholipid). Of note, masses of certain LCFs are not unique given that molecules from other lipid classes can release LCFs with identical masses (e.g. *m/z* 184.0733 can be released from LPC, PC, PC O-, SM species). Moreover, LCFs can only be used to identify lipid molecules at the “lipid species level” (e.g. PC 36:6).
2. MLFs (molecular lipid species-specific fragments) which are characterized by the properties that they can be both neutral and charged, and contain only one hydrocarbon chain with variations in the number of carbon atoms, double bonds and hydroxyl groups. Depending on the lipid class and category, these hydrocarbon chains can be classified as FA, alkanol and alkenol (i.e. plasmanyl and plasmenyl groups, respectively), LCB and sterol moieties (**S1 Fig**). MLFs can be used to identify lipid molecules at the “molecular lipid species level” (e.g. PC 18:3-13:3).
3. iMLFs (intermediate molecular lipid species-selective fragments) which are characterized by the properties that they can be both neutral and charged, and contain two or more hydrocarbon chains (e.g. DAG 36:6-containing fragments in **Fig 3**) that depending on the lipid class can be composites of FA, alkanol, alkenol, LCB and sterol moieties. As for LCFs, iMLFs can only be used to identify intact lipid molecules at the “lipid species level” (e.g. PE 36:2). Of note, charged iMLFs can be subject to MS^3^ fragmentation to yield MLFs that in turn enable identification of intact lipid molecules at the “molecular lipid species level” (e.g. PE 18:1-18:1).
4. Double bond location-specific fragments (DBFs) which are characterized by the properties that they can be both neutral and charged, and represent a fraction of a hydrocarbon chain produced by specific cleavage of a C-C double bond. An example of this fragment type includes the radical anion with *m/z* 182.1305 derived from FA 18:1(9) upon MS^3^ fragmentation of PC 16:0-18:1(9) (**Fig 4E** and **S3E Fig**). Together DBFs and MLFs can be used to identify lipid molecules at the “double bond location-defined molecular lipid species level” (e.g. PC 16:0-18:1(9)).

Examples of each of the four fragment types are highlighted in the fragmentation diagrams of representative lipid molecules shown in **Fig 3B** and **S3** **Fig**. We note that the abbreviation “LCF” (lipid class fragment) is chosen to emphasize the lipid molecule-specific origin of this type of fragments and distinguish these from fragments derived from other classes of metabolites. Similarly, the abbreviation “MLF” (molecular lipid species-specific fragment) is chosen to emphasize the direct link between this type of fragment and the appropriate shorthand notation to use for annotating an intact precursor lipid molecule.

**Definition of minimal hydrocarbon chain-based attributes (HCAs)**

For the purpose of formalizing a generic nomenclature for annotation of lipid fragment ions our systematic analysis also helped pinpoint that hydrocarbon chain-containing MLFs, iMLFs and DBFs can be described in reference to what we define as “minimal hydrocarbon chain-based attributes (HCAs)” (**S1 Fig**). HCAs represent the most basic unit of structural information carried by a given fragment (charged or uncharged) with respect to the variable hydrocarbon-based backbone of the intact precursor lipid molecule. Importantly, using minimal HCAs provides a mean to implement rules for annotating MLFs, iMLFs and DBFs using nomenclature that is similar to that of intact lipid molecules and as such it makes it more intuitive to correlate characteristics of lipid fragment ions back to the structures of intact lipid molecules.

Depending on lipid class, minimal HCAs can be classified as single hydrocarbon chains, including FA, LCB, alkanol and alkenol (i.e. plasmanyl and plasmenyl groups, respectively) or sterol residues, as structures having two hydrocarbon chains (e.g. DAG, Cer) and three hydrocarbon chains (e.g. MLCL). Examples of structures and shorthand notations of representative minimal HCAs classes are depicted in **S1 Fig**. We note that the FA (fatty acyl, R-CO), rather than the fatty acid (R-COOH), was chosen as a common HCA based on both biochemical considerations and lipid fragmentation behavior. These considerations includes the propensity of FA moieties to be esterified to glycero(phospho)lipids, sphingolipids and coenzyme A via oxygen, nitrogen and sulfur atoms, respectively. Moreover, fragmentation of sphingolipids in negative ion mode often concurs with molecular rearrangements where the amide-linked FA moiety is released as both amide and carboxylate anions having distinct *m/z* values. Hence, in our opinion it is more logical to annotate these fragments in reference to the FA (fatty acyl) moiety and not to the fatty acid (NEFA) (**Fig 4G** and **S3G Fig**).

**Rules for shorthand notation of different fragment types**

Having implemented a framework capable of recapitulating lipid fragmentation across different lipid categories and analytical conditions we next implemented rule sets for consistent shorthand notation of the different fragment types. These rule sets were designed to facilitate matching of fragment ion *m/z* values to structures of LCFs, MLFs, iMLFs and DBFs *that are released from a given precursor lipid upon fragmentation*. This strategy is different to that of other, non-formalized annotations where lipid fragment ion *m/z* values are typically denoted as ‘intact’ lipid molecules using shorthand notation such as “LPC 18:1-like” or using chemical formulas (e.g. C24H49NO6P) that makes it difficult to relate the putative structure of a fragment ion back to the structure of the intact molecule.

For the nomenclature we implemented the basic rule is that uncharged fragments should always be prefixed with a minus sign “-“ to indicate neutral loss, and charged fragments should be denoted without a minus sign. For multiply charged lipid precursor ions where structural attributes can be lost as charged entities we implemented the rule that these should be prefixed with a minus sign in parentheses “(-)”. For example, fragmentation of doubly charged cardiolipin (CL) results in loss of FA moieties as singly charged carboxylate anions producing singly charged fragments having structures reminiscent of dehydrated monolysocardiolipin (MLCL, **Fig 4D** and **S3D Fig**). Of note, for the sake of clarity and simplicity the nomenclature does not indicate the polarity of fragment ions as these are implicit from the acquired MS^n^ data. We note that we chose to use a minus sign “-“ to denote neutral fragments and no sign to denote charged fragments based the analogy in mathematics where a negative variable is denoted “-x” and a positive variable is denoted as “x”. As such, the mathematical concepts “positive” vs. “negative” are equivalent to “charged” vs. “neutral”, respectively.

Structures of LCFs can, as outlined above, be released from all molecules belonging to the same lipid class, and thus, provide information only about structural attributes that are common to that particular lipid class. Based on this commonality and the low level of structural information provided about intact lipid molecules we rationalized that annotation of LCFs should only be able to relay information about lipid class, chemical formula and the mass of a fragment. Based on biochemical considerations and also the history of lipidomics technology (e.g. many investigators know that “*m/z* 184” is diagnostic for PC species) we decided to implement the following two rules to annotate LCFs:

1. Neutral LCFs should be denoted with a minus sign followed by the lipid class abbreviation and its nominal mass in parentheses. For example, “-PS(87)” and “-PE(141)” are used to indicate neutral loss of serine and phosphoethanolamine from PS and PE lipids, respectively.
2. Charged LCFs should be denoted by the lipid class abbreviation followed by its nominal *m/z* in parentheses. For example, “PC(184)” and “SM(184)” are used to indicate the protonated phosphocholine fragment derived from PC and SM lipids, respectively.

The annotation of LCFs from lipids spanning different lipid categories is exemplified in **Figs 3 and 4** and **S3 Fig**. An alternative approach to denote LCFs could be to use their chemical formula. However, when using this style of annotation the PC 18:3-18:3-derived fragment ion with *m/z* 762.5079 should be annotated as “C43H73NO8P” instead of “-PC(74)” (**Fig 3**). In our opinion using chemical formula for shorthand notation makes it unnecessary difficult to correlate fragment ion *m/z* values to structures of fragments and their origin. We note, however, that chemical formula for fragment ions can easily be stored as metadata in fragmentation databases (see section on ALEX^123^ lipid calculator in the main text).

For shorthand notation of highly variable MLFs we rationalized that it would be advantageous to implement a nomenclature that would relay whether fragments are structurally related to FA, alkanol, alkenol, LCB and sterol moieties, and also communicate the number of carbon atoms, double bonds and potential hydroxyl groups in the hydrocarbon chain from which they derive. Using the concept of minimal HCAs, and also systematically evaluating structures of MLFs across five different categories of lipids, we found that these fragments can be consistently annotated using the following two rules:

1. Neutral MLFs should be denoted with a minus sign followed by the class of HCA, its original number of carbon atoms, double bonds and potential hydroxyl groups, and followed by parentheses with specification of any chemical modification relative to the structure of the minimal HCA. Any chemical modification attributed additions and subtractions relative to the minimal HCA should be specified with plus “+” and minus “-” signs followed by atomic differences listed in accordance to Hill notation. For example, “-FA 18:3(+HO)” and “-FA 18:3(-H)” are used to indicate neutral loss of FA 18:3 moiety as a fatty acid and a ketene, respectively (**Fig 3**).
2. Charged MLFs should be denoted by the class of HCA, number of carbon atoms, double bonds and potential hydroxyl groups, followed by specification of any chemical modifications relative to the minimal HCA in parentheses. Any chemical modification attributed additions and subtractions relative to the minimal HCA should be specified with plus “+” and minus “-” signs followed by atomic differences listed in accordance to Hill notation. For example, “FA 17:0(+NH)”, ”FA 17:0(+O)” and “FA 17:0” are used to indicate a FA 17:0 moiety released as an amide anion, as a carboxylate anion and as a positive acylium, respectively (**Fig 4G** and **S3G Fig**).

Examples of annotated MLFs are shown in the fragmentation diagrams shown in **Fig 3B** and **S3 Fig**. An alternative nomenclature could be to denote MLFs by their chemical formula. As such, the PC 18:3-18:3 derived MLF ion with *m/z* 502.2940 (**Fig** **3A**) would be annotated as “C26H47NO6P” instead of “-FA 18:3(+HO) -PC(74)”. In our opinion using this alternative style of annotation makes it difficult to correlate annotated fragment ion *m/z* values back to the structures of intact lipid molecules. Hence, we argue that it is much more informative to denote MLFs using a nomenclature that communicates the putative structure of the underlying HCA and intact lipid molecule.

To annotate iMLFs we implemented a rule set similar to that of MLFs. This rationale was based on systematically inspecting structures of iMLF released from the different lipid molecules included in our study. This analysis revealed that iMLFs could be characterized as both neutral and charged structures having two or three hydrocarbon chains and featuring various lipid class-dependent chemical modifications. Based on this we implemented the following two rules for annotating iMLFs:

1. Neutral iMLFs should be denoted with a minus sign followed by the class of HCA, its original number of carbon atoms, double bonds and potential hydroxyl groups, and followed by in parentheses any chemical modifications relative to the structure of the minimal HCA. Any chemical modification attributed additions and subtractions relative to the minimal HCA should be specified with plus “+” and minus “-” signs followed by atomic differences listed in accordance to Hill notation. For example, “-DAG 36:6(-H2O)” is used to indicate neutral loss of a dehydrated DAG 36:6 moiety (**Fig 3B**).
2. Charged iMLFs should be denoted by the class of HCA, its original number of carbon atoms, double bonds and potential hydroxyl groups, and followed by in parentheses any chemical modification relative to the structure of the minimal HCA. Any chemical modification attributed additions and subtractions relative to the minimal HCA should be specified with plus “+” and minus “-” signs followed by atomic differences listed in accordance to Hill notation. For example, “Cer 35:1;2(-HO)” is used to indicate a dehydrated and charged Cer 35:1;2 moiety (**Fig 4F** and **S3F Fig**, *m/z* 534.5245).

Examples of annotated iMLFs are shown in fragmentation diagrams shown in **Fig 3B** and **S3 Fig**. A particular well-suited example for showcasing the annotation of iMLF-based structures is the fragmentation of double deprotonated and negatively charged CL 14:1-14:1-14:1-15:1 (**Fig 4D** and **S3D Fig**). Its fragmentation yields a fragment ion with *m/z* 1019.5977 that can be explained by loss of an FA 14:1 moiety as singly charged carboxylate anion (denoted as (-)FA 14:1(+O), an MLF) producing a charged iMLF with a structure reminiscent of a dehydrated MLCL having 43 carbon atoms and 3 double bonds in its remaining FA moieties (denoted as MLCL 43:3(-H3O)). Similarly, the fragment ion with *m/z* 1005.5824 can be explained by the loss of FA 15:1 moiety as a singly charged carboxylate anion (denoted as (-)FA 15:1(+O), a MLF) producing a charged iMLF with a structure reminiscent of dehydrated MLCL having a total of 42 carbon atoms and 3 double bonds in its FA moieties (denoted as MLCL 43:3(-H3O)). The fragmentation also results in neutral loss of FA moieties as ketenes (e.g. -FA 14:1(-H), a MLF) producing doubly charged iMLFs with *m/z* 511.2923 and having a structure reminiscent of doubly deprotonated MLCLs (denoted for example as MLCL 43:3(-H2)). Interestingly, the fragmentation of the CL molecule also results in the fragment ion with *m/z* 643.3965 corresponding to the loss of a singly charged iMLF having a DAG 29:2 backbone linked to a phosphoryl group (denoted as (-)DAG(+O3P)) producing a singly charged iMLF structure having a DAG 28:2 backbone with a dehydrated phosphoglyceryl group (denoted as DAG 28:2(+C3H4O4P)). Of note, using non-formalized annotation this fragment ion *m/z* value would be denoted as “PG 28:2-like”. Similarly, loss of the singly charged iMLF DAG 28:2(+C3H4O4P) (now annotated as (-)DAG 28:2(+C3H4O4P)) produces the fragment ion with *m/z* 601.3864 which corresponds to a singly charged iMLF having a DAG 29:2 backbone linked to a phosphoryl group (now denoted as DAG(+O3P)). These non-exhaustive examples of iMLFs and their systematic annotation demonstrate that our nomenclature rules are able to effectively handle lipid molecules with more complicated fragmentation behaviors, such as CL molecules.

To annotate DBFs we implemented a rule set similar that of MLFs and iMLFs with the only exception that minimal HCA in this case should include nomenclature for specifying the locations of carbon-carbon double bonds. Given that DBFs can be both neutral and charged we defined the following two rules for annotating these:

1. Charged DBFs should be denoted by the class of HCA, its original number of carbon atoms, number of double bonds and locations of double bonds, followed by in parentheses any chemical modification relative to the structure of the minimal HCA. Any chemical modification attributed additions and subtractions relative to the minimal HCA should be specified with plus “+” and minus “-” signs followed by atomic differences listed in accordance to Hill notation. For example, “FA 18:1(9)(+O -C7H15)” is used to indicate the neutral loss of the radical C7H15 from the FA 18:1(9) moiety (**Fig 4E** and **S3E Fig**).
2. Neutral DBFs should be denoted with a minus sign followed by the class of HCA, its original number of carbon atoms and double bonds, and locations of double bonds followed by in parentheses any chemical modifications relative to the structure of the minimal HCA. Any chemical modification attributed additions and subtractions relative to the minimal HCA should be specified with plus “+” and minus “-” signs followed by atomic differences listed in accordance to Hill notation. For example, “-FA 18:1(9)(-C11H18O)” is used to indicate the neutral loss of the radical C11H18O from the FA 18:1(9) moiety (**Fig 4E** and **S3E Fig**).

Interestingly, detection of a double bond location-specific radical anion by MS^3^ analysis of PC 16:0-18:1(9) (and other lipids, data not shown) on an Orbitrap Fusion mass spectrometer represents a novel and simple avenue for identification of lipids at “double bond location-defined molecular lipid species level”. Notably, this radical anion is detected together with a complementary fragment ion having a nominal mass difference of +1 Da (**Fig 4E** and **S3E Fig**).

**Rules for shorthand notation of fragment ions in mass spectra**

Given that fragment ion *m/z* values can be ascribed to two redundant annotations, corresponding to either a charged fragment structure or the composite of neutral fragment structures, we implemented the following rules for shorthand hand notation of fragment ion *m/z* values in mass spectra of lipid molecules (summarized in **Fig 2**):

1. Shorthand notation of a particular fragment ion *m/z* value should be prioritized according to fragment type in the following order: DBFs, MLFs, LCFs and iMLFs. For example, a fragment ion *m/z* value with double bond-specific information should be annotated with nomenclature according to DBFs instead of, for example, nomenclature based on iMLFs or composites of fragment types. Similarly, a fragment ion *m/z* value featuring MLF information, for example, “-FA 18:1(+HO) -TAG(17)” from TAG 54:3 should be prioritized over the iMLF information “DAG 36:2(-HO)” (**Fig 4B** and **S3B Fig**). A fragment ion *m/z* value featuring LCF information, for example, “SM(184)” from intact SM 35:1;2 should be prioritized over the iMLF information “-Cer 35:1;2(-H2O)” (**Fig 4F** and **S3F Fig**).
2. Shorthand notation of fragment ion *m/z* values corresponding to DBFs should primarily be based on nomenclature of the charged fragment structure, and secondarily based on neutral fragment structure(s).
3. Shorthand notation of fragment ion *m/z* values corresponding to MLFs should be prioritized according to the fragment structure(s), either the charged fragment or the composites of neutrals, that has the lowest mass (not *m/z*). For example, the fragment ion with *m/z* 361.2704 released from PE O-18:1p/20:4 (**Fig 4C**) can be annotated as a charged MLF “FA 20:4(+C3H5O2)” with mass 361 Da and as a neutral composite of an MLF and an LCF “-18:1p -PE O-(123)” with a total mass of 391 Da. In this case the annotation based on the charged structure is prioritized (i.e. “FA 20:4(+C3H5O2)”).
4. Shorthand notation of fragment ion *m/z* values corresponding to LCFs should primarily be based on the nomenclature of charged fragment structure, and secondarily based on neutral fragment structure(s).
5. Shorthand notation of fragment ion *m/z* values corresponding to iMLFs should be prioritized according to the fragment structure(s), either the charged fragment or the composites of neutrals, that has the lowest mass (not *m/z*). For example, the fragment ion with *m/z* 657.4123 released from CL 14:1-14:1-14:1-15:1 (**Fig 4D** and **S3D Fig**) can be annotated as a charged iMLF “DAG 29:2(+C3H4O4P)” with mass 657 Da or a loss of a charged iMLF “(-)DAG 28:2(+O3P)” with mass 587 Da. In this case the annotation based on the loss of the charged iMLF is prioritized (i.e. “(-)DAG 28:2(+O3P)”).

An overview of the procedure for constructing and prioritizing rules for shorthand notation of fragment ion *m/z* values in mass spectra of lipids is outlined in **Fig 2**.

We note that the nomenclature might, for some investigators, appear counterintuitive at times. For example, CID of ammoniated TAG 18:0-18:1-18:2 produces the fragment ion with *m/z* 263.2374 which matches a FA 18:2 acylium that is denoted without using parentheses as “FA 18:2”. This shorthand notation is sometimes used to denote FA carboxylate anions using non-formalized nomenclature. However, following the nomenclature rule for MLFs (R2.2) outlined above in the previous section this shorthand notation is consistently implemented as no chemical modification is present relative to the defining minimal HCA (i.e. FA 18:2). Conversely, applying rule R2.2 the FA carboxylate anion at *m/z* 277.2172 released from PC 18:3-18:3 should be annotated as “FA 18:3(+O)” to express that the minimal HCA is an FA 18:3 carrying an additional oxygen atom. In addition, CID of FA carboxylate anions often produces fragment ions with 18.0106 Da and 36.0211 lower mass that corresponds to loss of one and two H_2_Os, respectively. This issue is exemplified for the MS^3^ analysis of the FA 18:1 fragment ion derived from PC 16:0-18:1(9) (**Fig 4E** and **S3E Fig**) which produces fragment ions with *m/z* 263.2376 and *m/z* 245.2268. Based on nomenclature rules R2.2 and R5.1 these *m/z* values should be annotated as “FA 18:1(-H)” and “FA 18:1(-H3O)”. This might appear some inconsistent. However, we argue that proposed nomenclature rules are consistently implemented, are applicable to fragmentation in both positive and negative ion mode, and are independent of lipid class as long as any chemical addition/abstraction to a MLF (or iMLF and DBF) is specified *relative* to the structure of the minimal HCA. An alternative could be to devise a nomenclature based on annotation rules that are specific for polarity and individual lipid classes. This alternative would in our opinion be much more inconsistent, cumbersome to implement and more difficult standardize (which we have tried to do).

**Rules for annotating intact stable isotope-labeled lipids and their fragments.**

As a first step to make the lipid fragment ion nomenclature applicable to stable isotope-labeled lipid molecules we adapted the guidelines for shorthand notation of intact lipid molecules at the “lipid species level”. To the existing guidelines we added the rule that intact lipid species labeled with stable isotopes should be named using the following convention:

1. The nomenclature for shorthand notation of lipid species should be followed by in parentheses a “+” sign, the heavy nuclei indicated by their isotope number in squared brackets, their atomic symbol and their count, listed in accordance to Hill notation. For example, a PC 34:1 molecule labeled with three ^13^C atoms and nine ^2^H atoms should be annotated as PC 34:1(+[13]C3[2]H9).

This nomenclature does not provide any information as to where in the lipid molecule the stable isotopes are positioned. Hence, we extended the rule set to also include shorthand notation at the “molecular lipid species level”. To this end, we extended the annotation rules described in the sections above (R6.1) and implemented the following rule:

1. The above-defined naming convention for heavy nuclei (R6.1, e.g. “+[13]C2[15]N”) should follow either the abbreviation of the lipid class or the HCA into which it is incorporated. For example, the lipid species PC 34:1(+[13]C5[2]H9) with nine ^2^H atoms in the head group and five ^13^C atoms in the 18:1 moiety should be annotated as PC(+[2]H9) 16:0-18:1(+[13]C5).

Next we extended the rule set for annotation of lipid fragment ions to also accommodate the presence of heavy nuclei. These rule sets were extended as:

1. LCFs containing heavy nuclei should be denoted according R1.1 and R1.2 with the insertion of the above-defined naming convention for heavy nuclei (R6.1) after the lipid class abbreviation. For example, a phosphocholine fragment ion containing nine ^2^H atoms and with *m/z* 197.1549 should be named as “PC(+[2]H9)(197)” (**Fig 7A**).
2. MLFs with heavy nuclei should be named according to R2.1 and 2.2 with the insertion of the above-defined naming convention for heavy nuclei (R6.1) after the abbreviation of the HCA moiety. For example, a neutral loss of a FA 16:0 moiety containing three ^2^H atoms as a ketene should be named as “-FA 16:0(+[2]H3)(-H)” (**Fig 7B**).

To exemplify how the nomenclature is practically implemented we show in **Fig 7** representative FTMS^2^ spectra of lipid molecules labeled with stable isotopes. For LCFs, this figure shows, for example, that “-PC(+[2]H13)(196)” and “PC(+[2]H13)(197)” indicate a neutral and a charged phosphocholine structure labeled with thirteen ^2^H atoms, respectively (**Fig 7A**). Similarly, **Fig 7C** shows “PI(+[2]H6)(229)” and “PI(+[2]H6)(247)” as examples of LCFs derived from a PI molecule labeled with six ^2^H atoms in the inositol head group.

For MLFs, **Fig 7B** shows the fragment ions annotated as “-FA 16:0(+[2]H3)(+HO)” and “-FA 16:0(+[2]H3)(-H)” which indicates neutral loss of an FA 16:0 moiety having three ^2^H atoms as an acid and as a ketene, respectively. Similarly, **Fig 7D** shows LCB 18:0;3(+[13]C2[15]N)(-H) and LCB 18:0;3(+[13]C2[15]N)(-[13]CH8[15]NO) as examples of MLFs derived from a Cer molecule having two ^13^C atoms and one ^15^N atom incorporated into its LCB moiety.
