# Supplementary material for: Proposal for a common nomenclature for fragment ions in mass spectra of lipids
Source: PLoS One. 2017 Nov 21;12(11):e0188394. doi: 10.1371/journal.pone.0188394 (PMC5697860; doi:10.1371/journal.pone.0188394)
Supplement: S1 Fig — (PDF) [file pone.0188394.s003.pdf]

S1 Fig

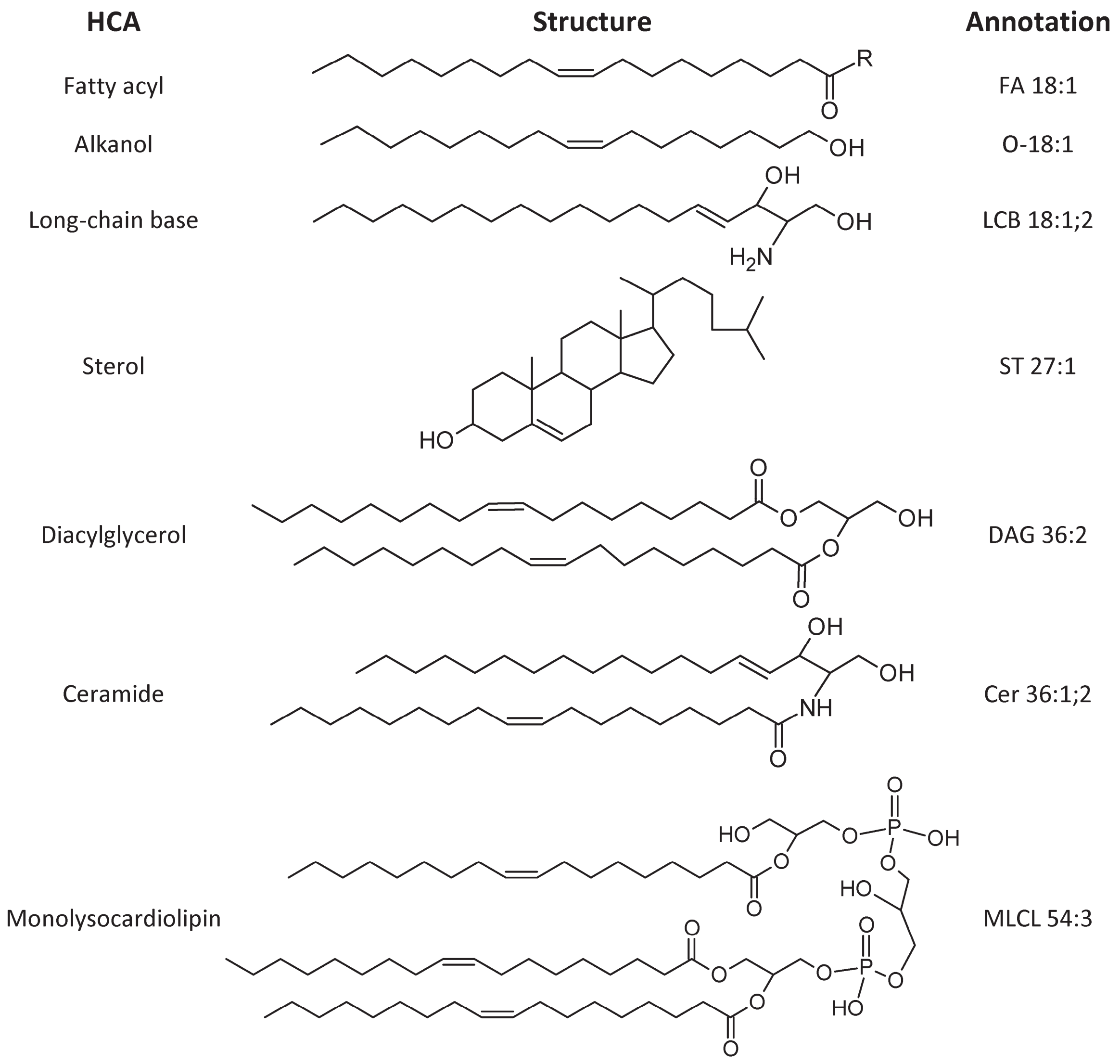

S1 Fig. Examples of minimal HCAs (hydrocarbon chain-based attributes) used for annotating neutral and charged fragment structures. As described in the text, fragment structures containing HCAs are referenced by using the annotation of HCAs followed by chemical modifications in parentheses (e.g. “FA 18:1(+O)” denotes an acyl carboxylate anion). To eliminate possible inconsistencies due to differences in polarity, charge states and adducts, all fragment structures are annotated relative to neutral HCAs.
