# Supplementary material for: Proposal for a common nomenclature for fragment ions in mass spectra of lipids
Source: PLoS One. 2017 Nov 21;12(11):e0188394. doi: 10.1371/journal.pone.0188394 (PMC5697860; doi:10.1371/journal.pone.0188394)

S2A Fig) -FTMS^2 *m/z* 871.5, [PI 17:0-20:4 -H]$^-$

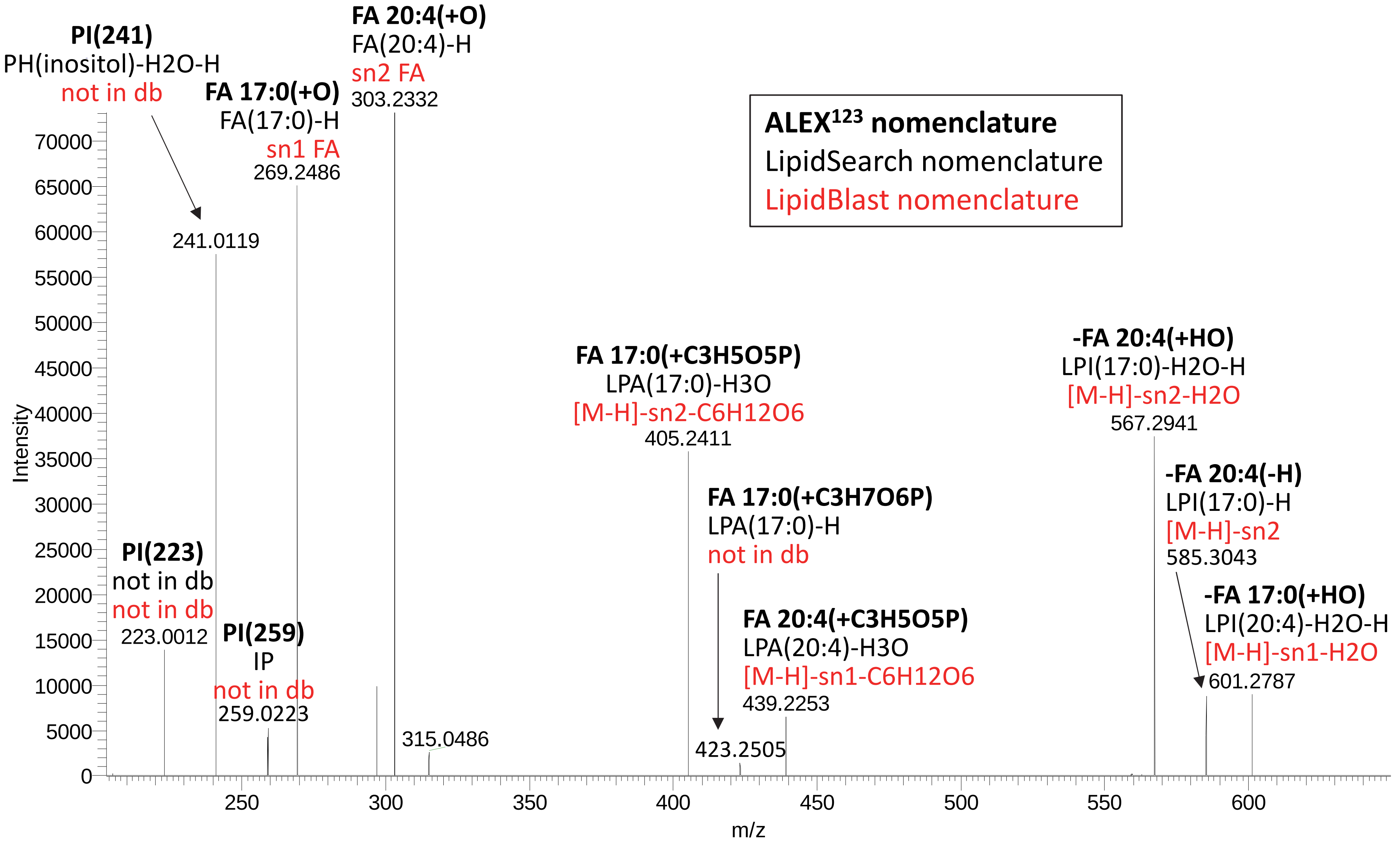

S2B Fig) -FTMS^2 *m/z* 796.5, [PS 17:0-20:4 -H]$^-$

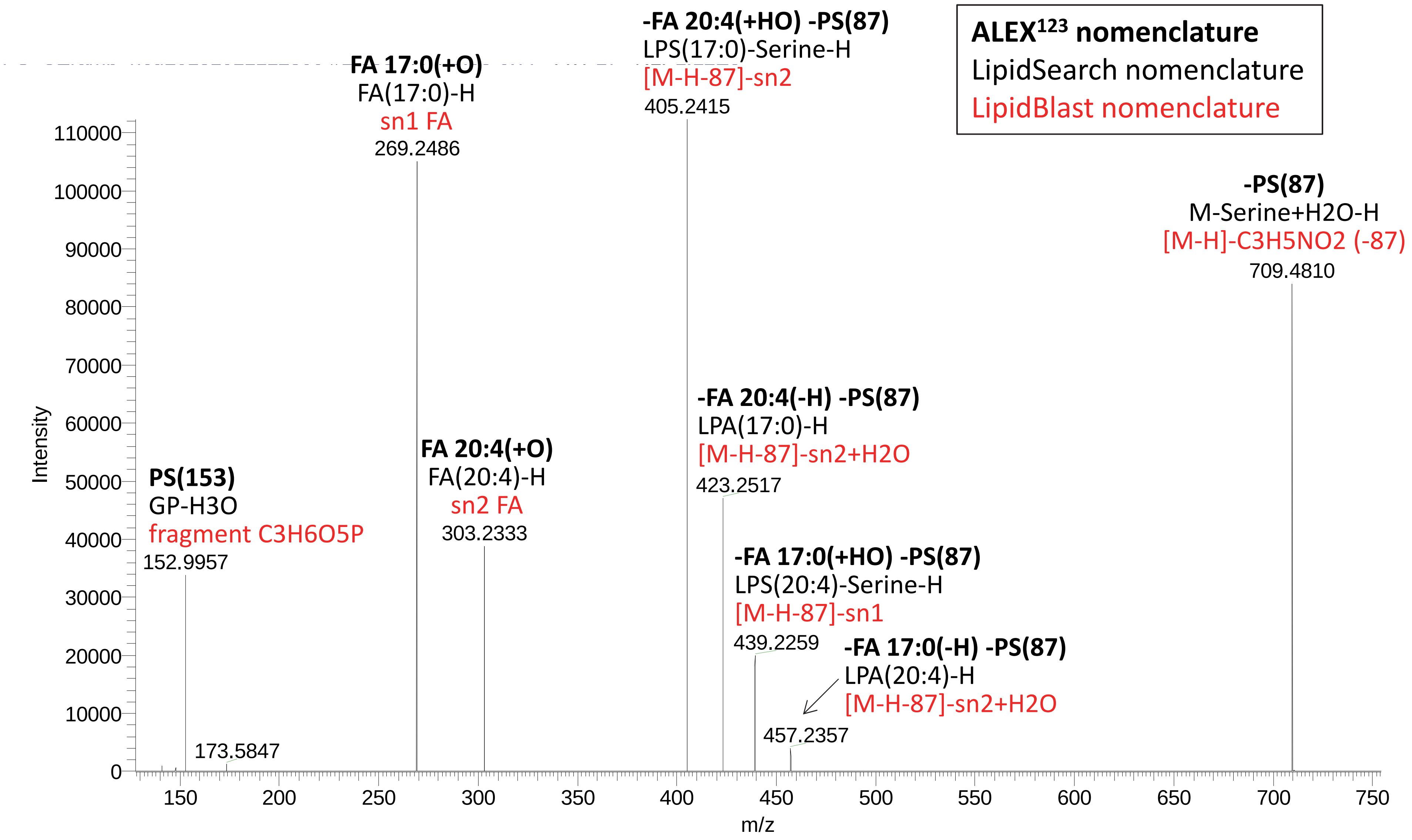

S2C Fig) +FTMS^2 *m/z* 644.5, [GlcCer 18:1;2/12:0 +H]$^+$

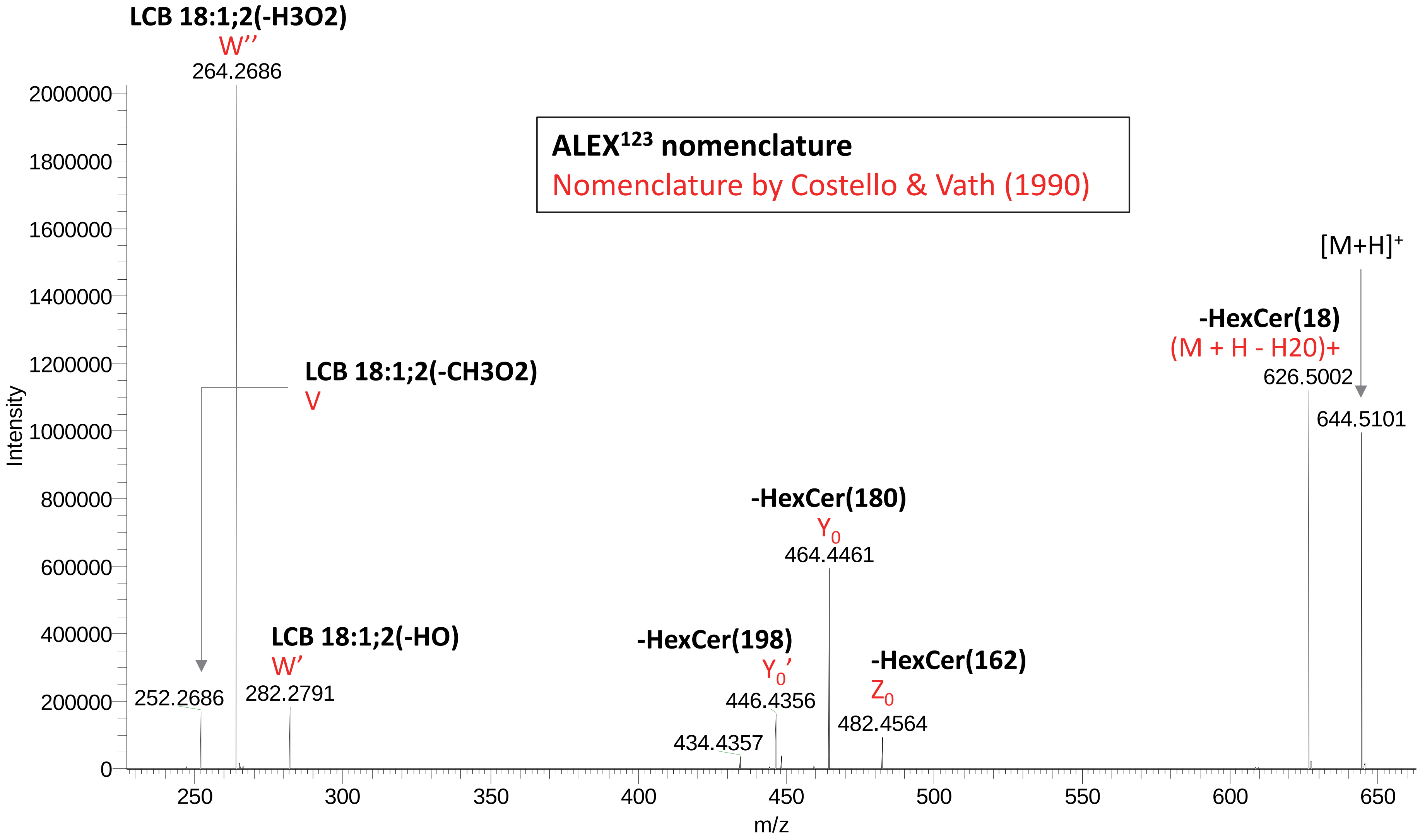

Supplement: S2 Fig — (PDF) [file pone.0188394.s004.pdf]
