# Supplementary material for: Proposal for a common nomenclature for fragment ions in mass spectra of lipids
Source: PLoS One. 2017 Nov 21;12(11):e0188394. doi: 10.1371/journal.pone.0188394 (PMC5697860; doi:10.1371/journal.pone.0188394)

S3A Fig) ACoA 19:0

Proposed structures corresponding to spectrum shown in Fig. 3A.

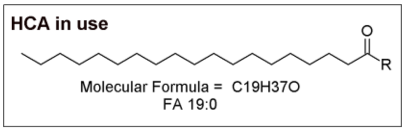

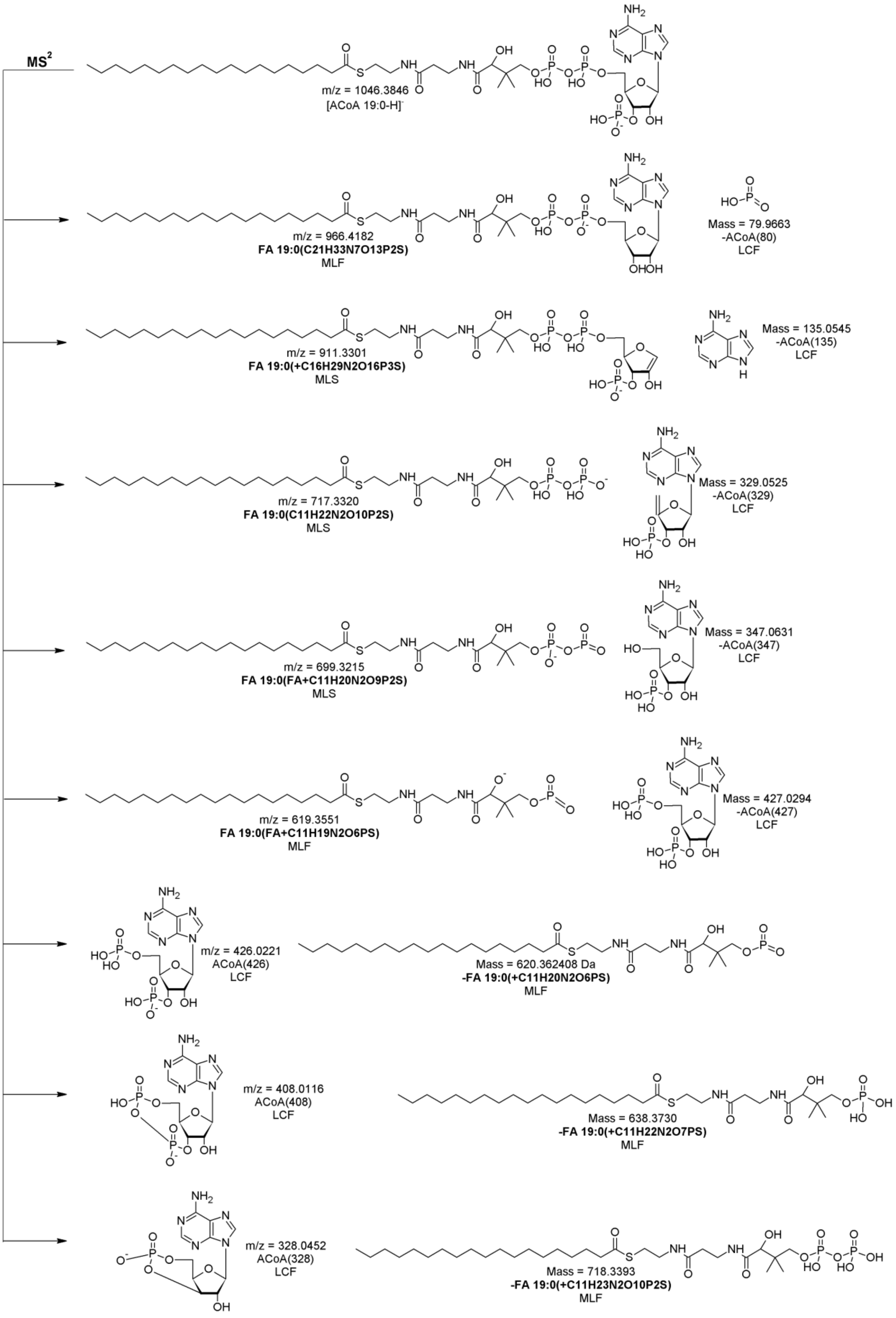

S3B Fig) TAG 18:0-18:1-18:2

Proposed structures corresponding to spectrum shown in Fig. 3B.

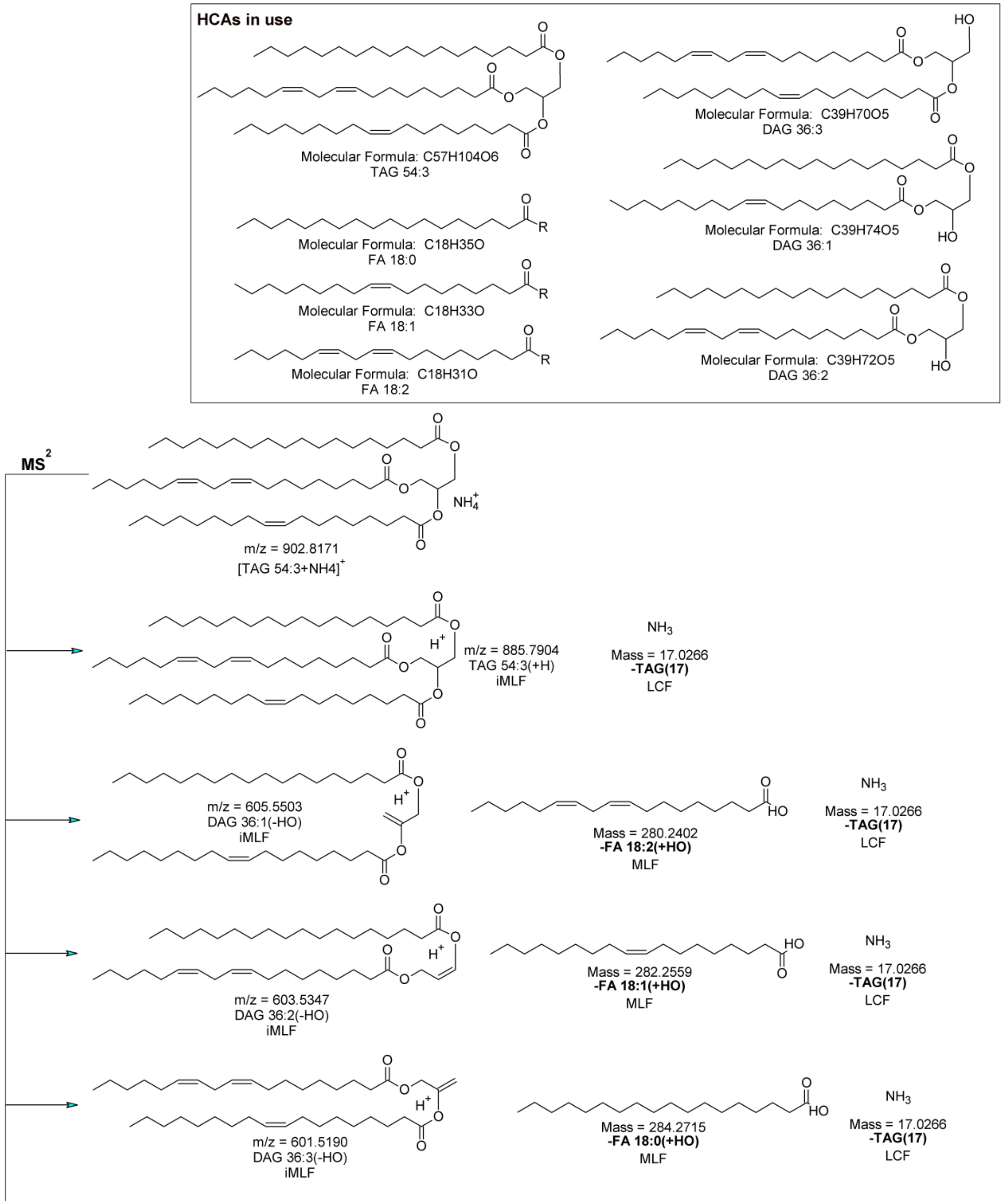

S3B Fig) TAG 18:0-18:1-18:2 (cont.)

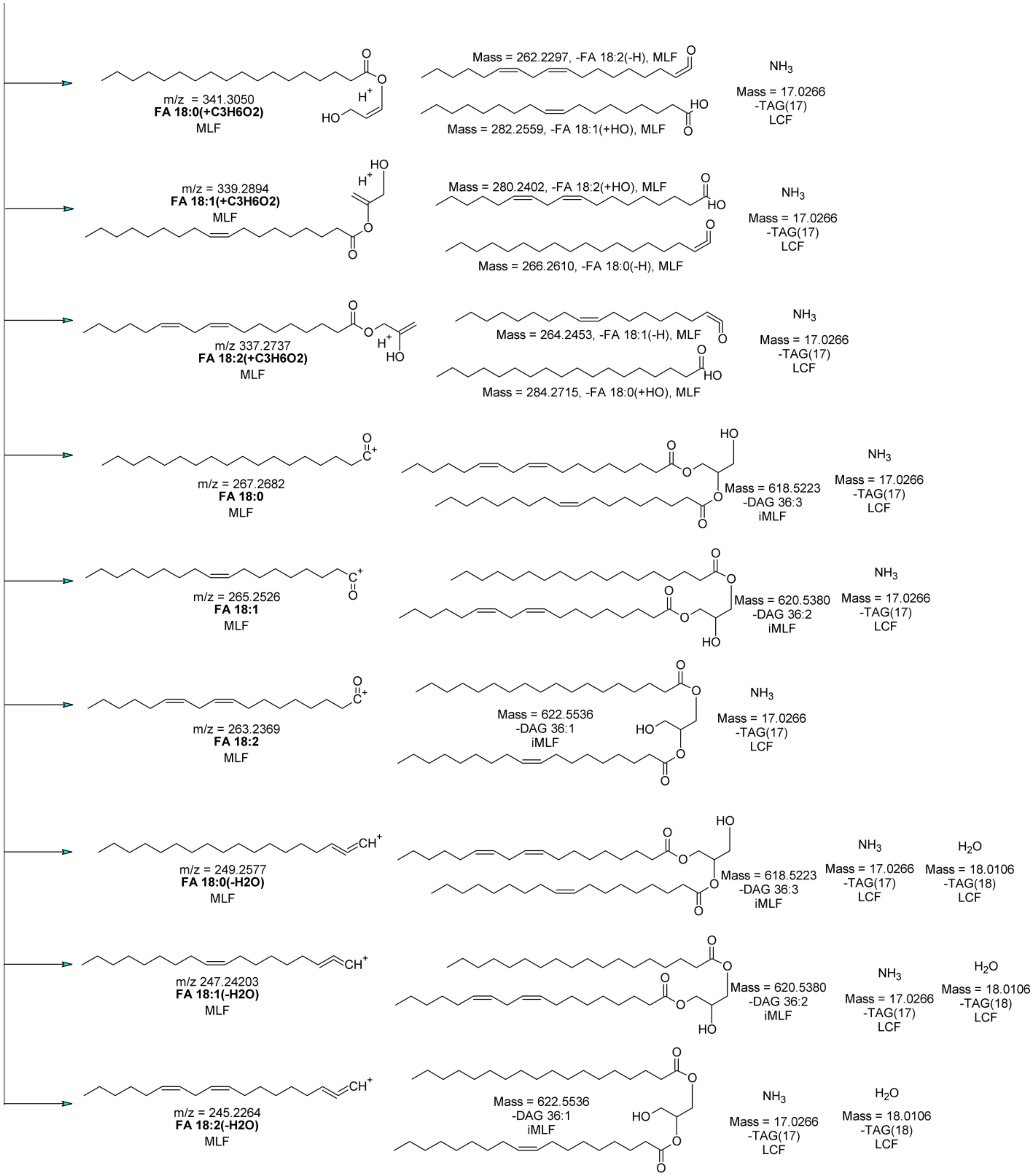

S3C Fig) PE O-18:1p/20:4

Proposed structures corresponding to spectrum shown in Fig. 3C.

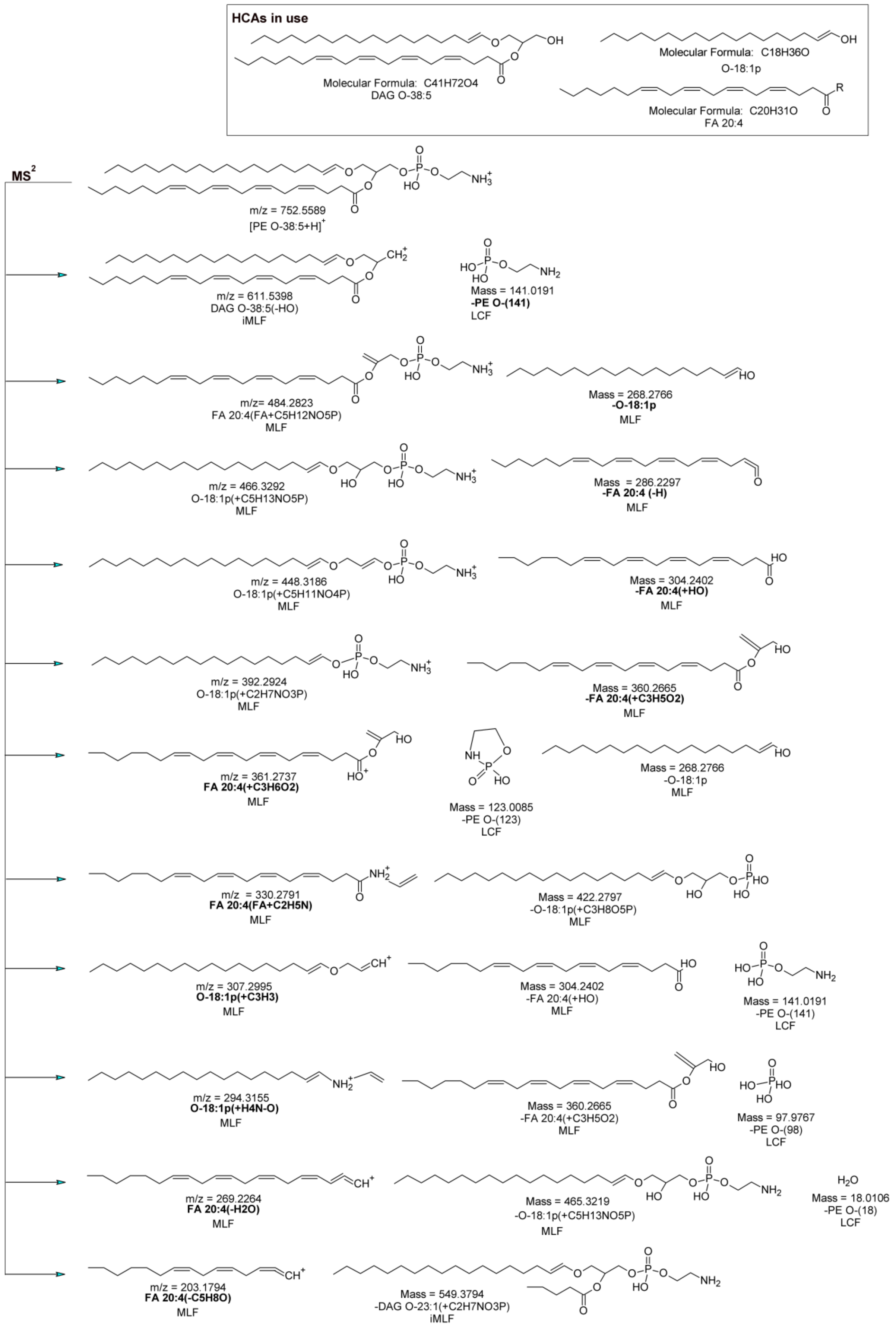

S3D Fig) CL 14:1-14:1-14:1-15:1

Proposed structures corresponding to spectrum shown in Fig. 3D.

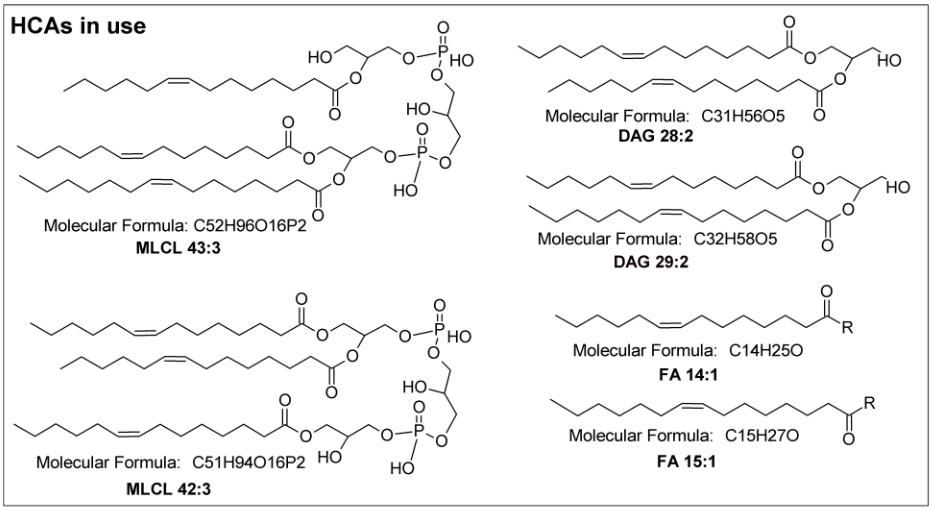

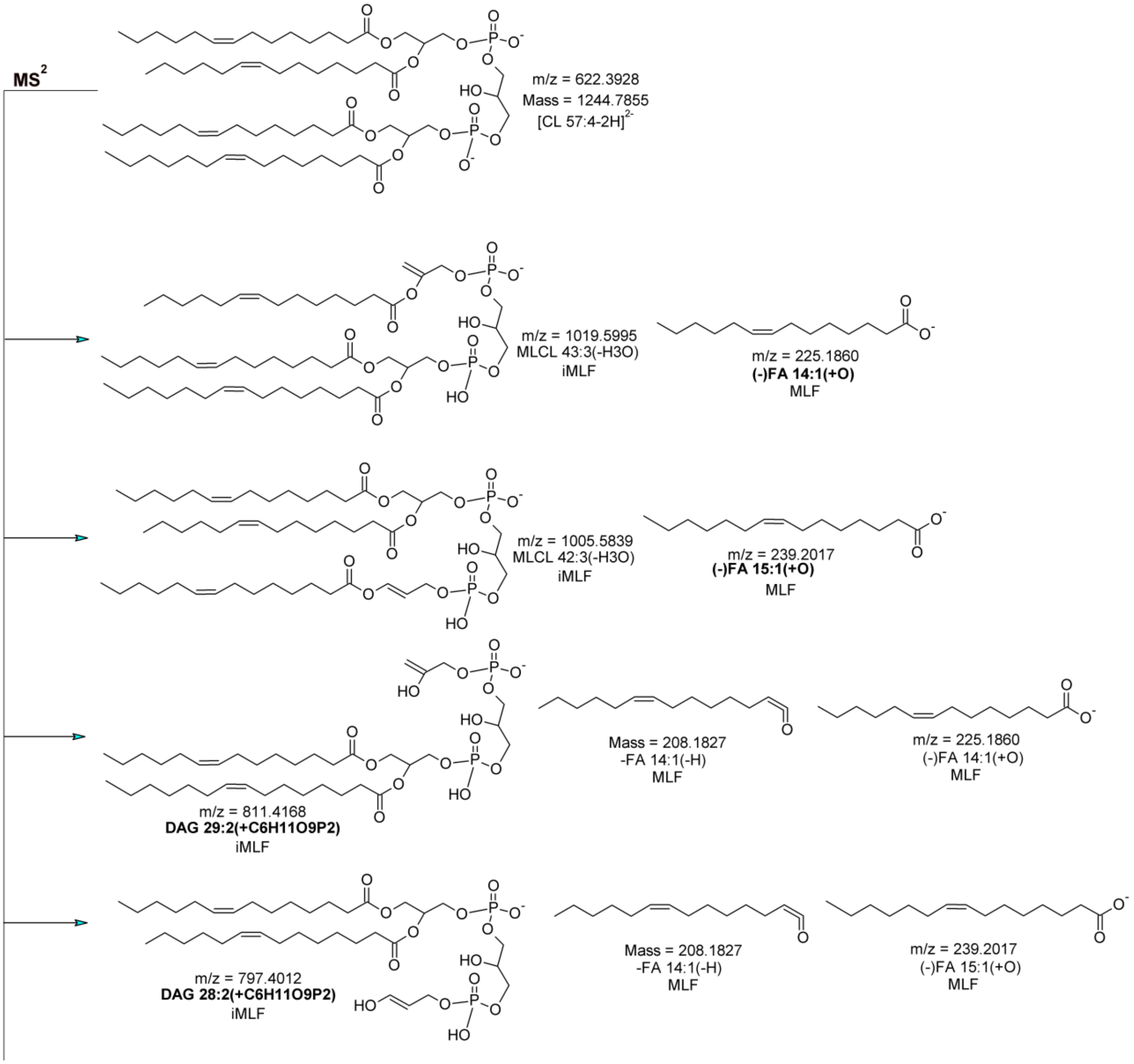

S3D Fig) CL 14:1-14:1-14:1-15:1 (cont.)

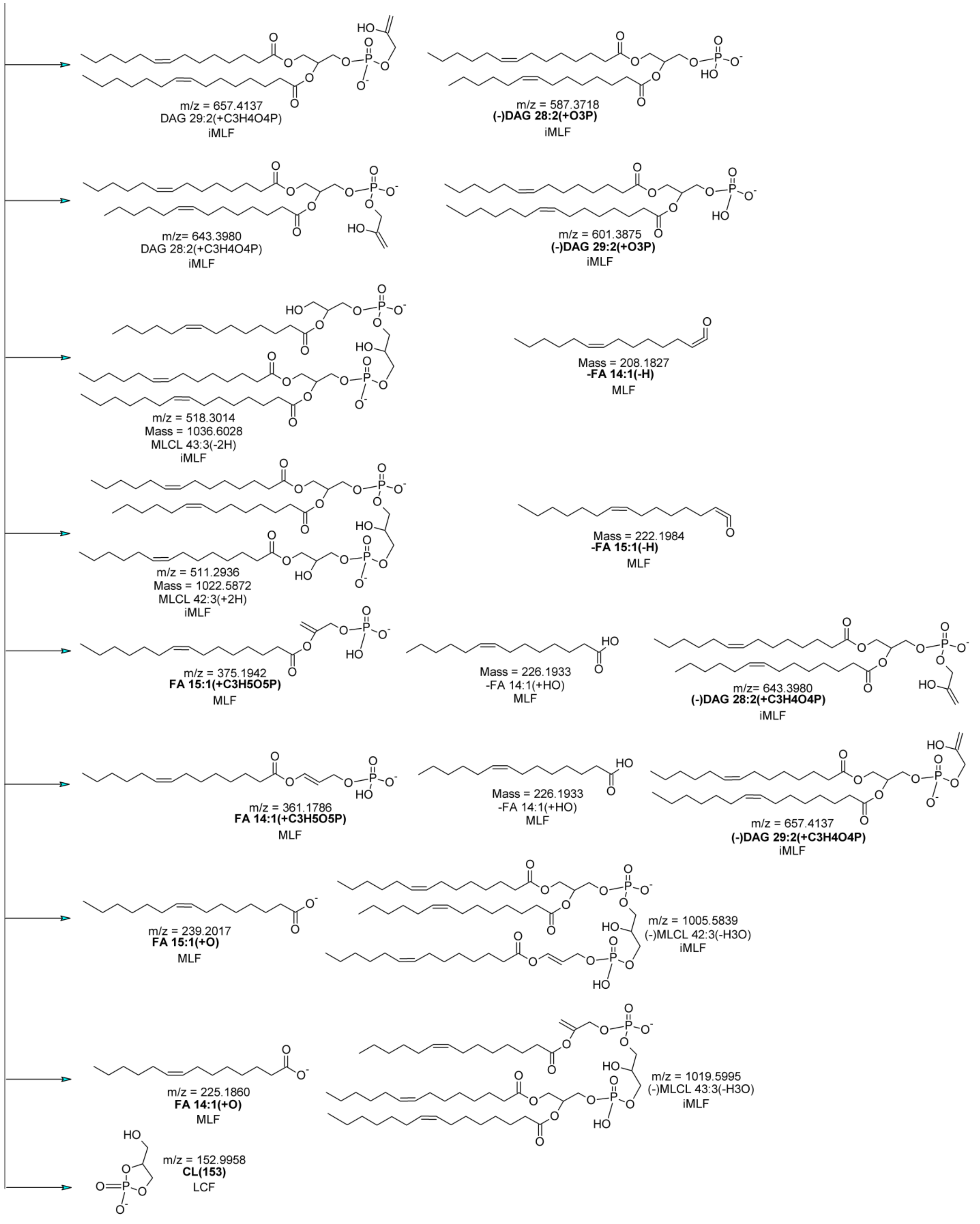

S3E Fig) PC 16:0-18:1(9)

Proposed structures corresponding to spectrum shown in Fig. 3E.

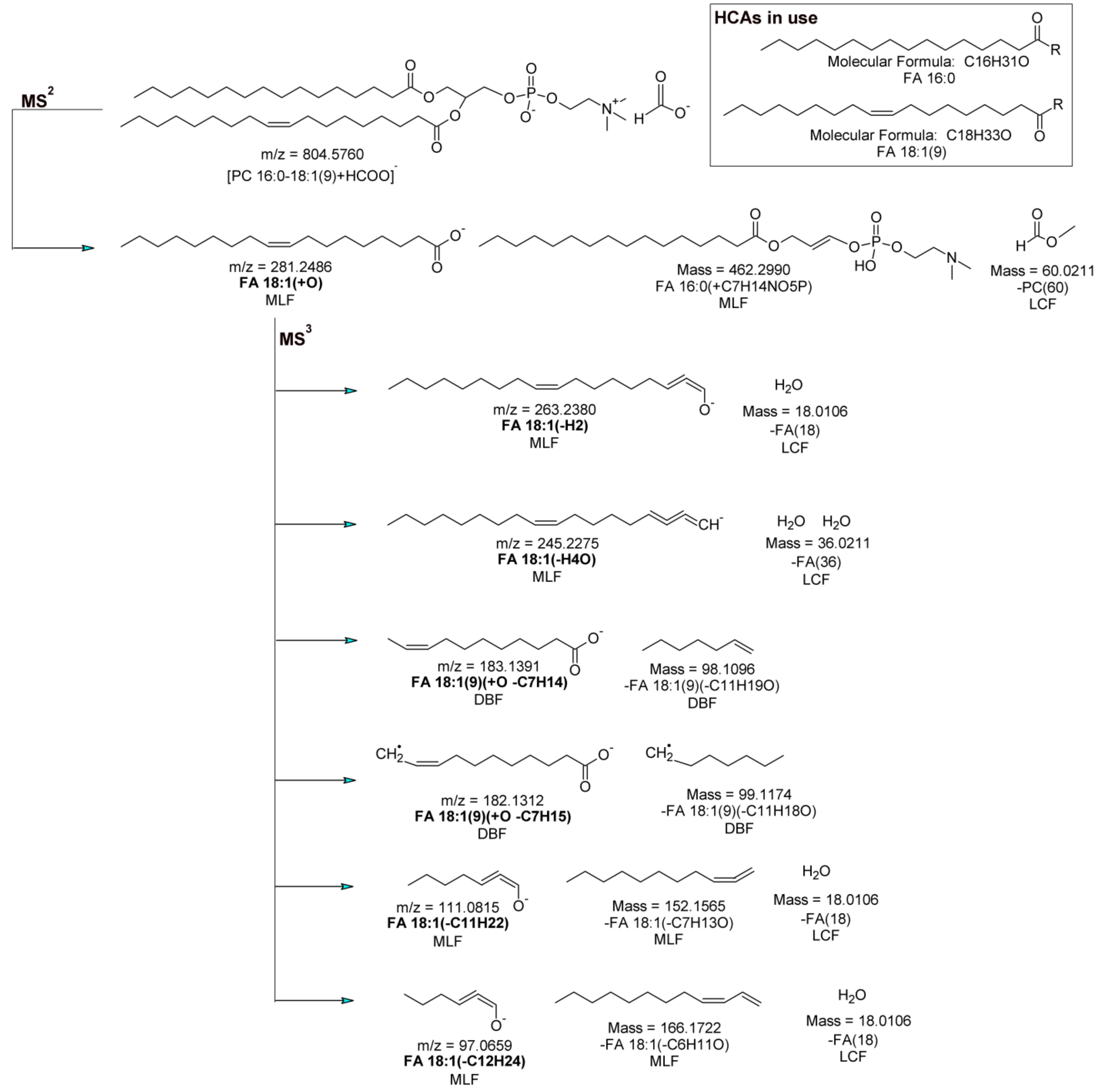

S3F Fig) SM 18:1;2/17:0

Proposed structures corresponding to spectrum shown in Fig. 3F.

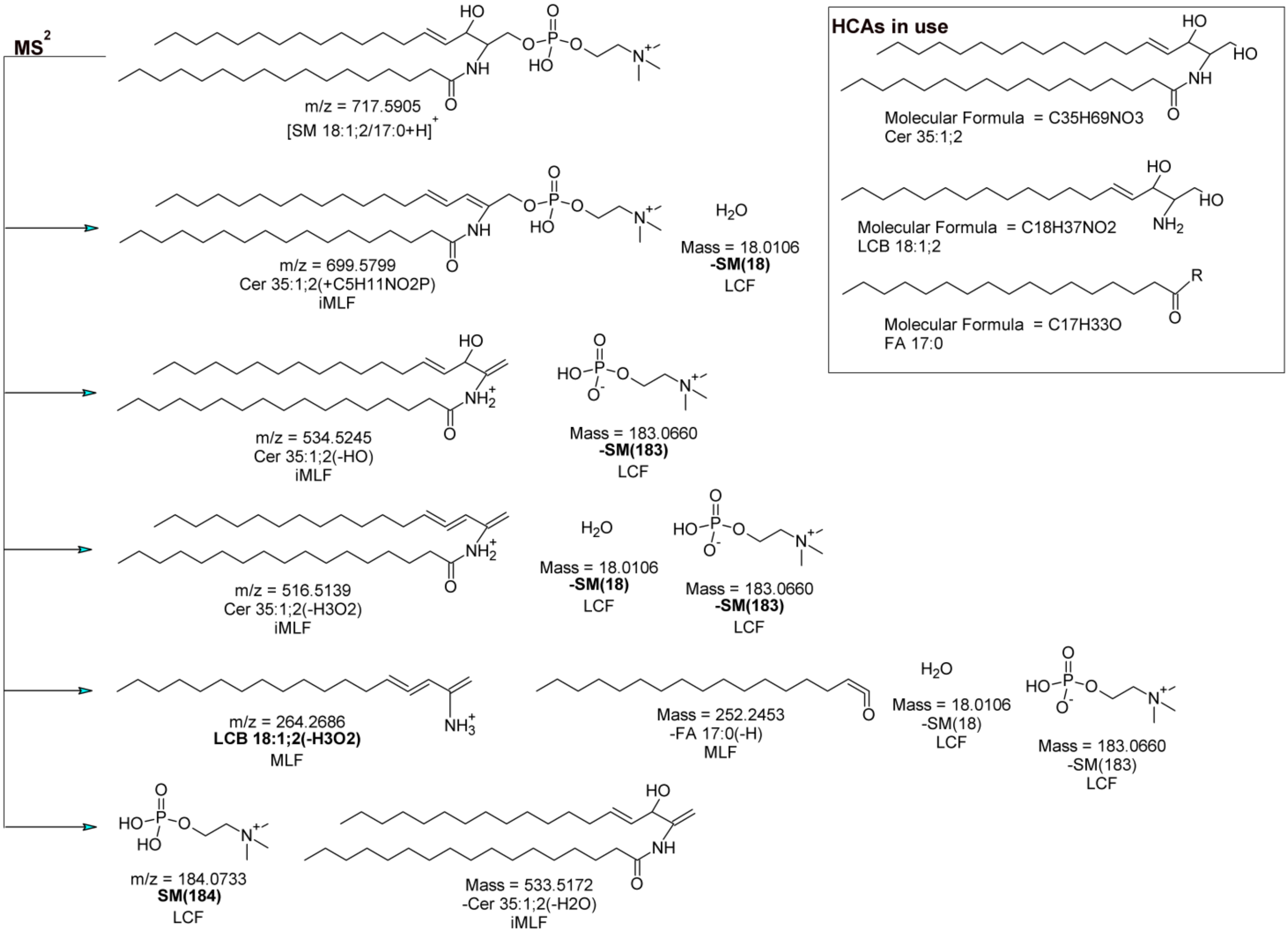

S3G Fig) Cer 18:1;2/17:0;1

Proposed structures corresponding to spectrum shown in Fig. 3G.

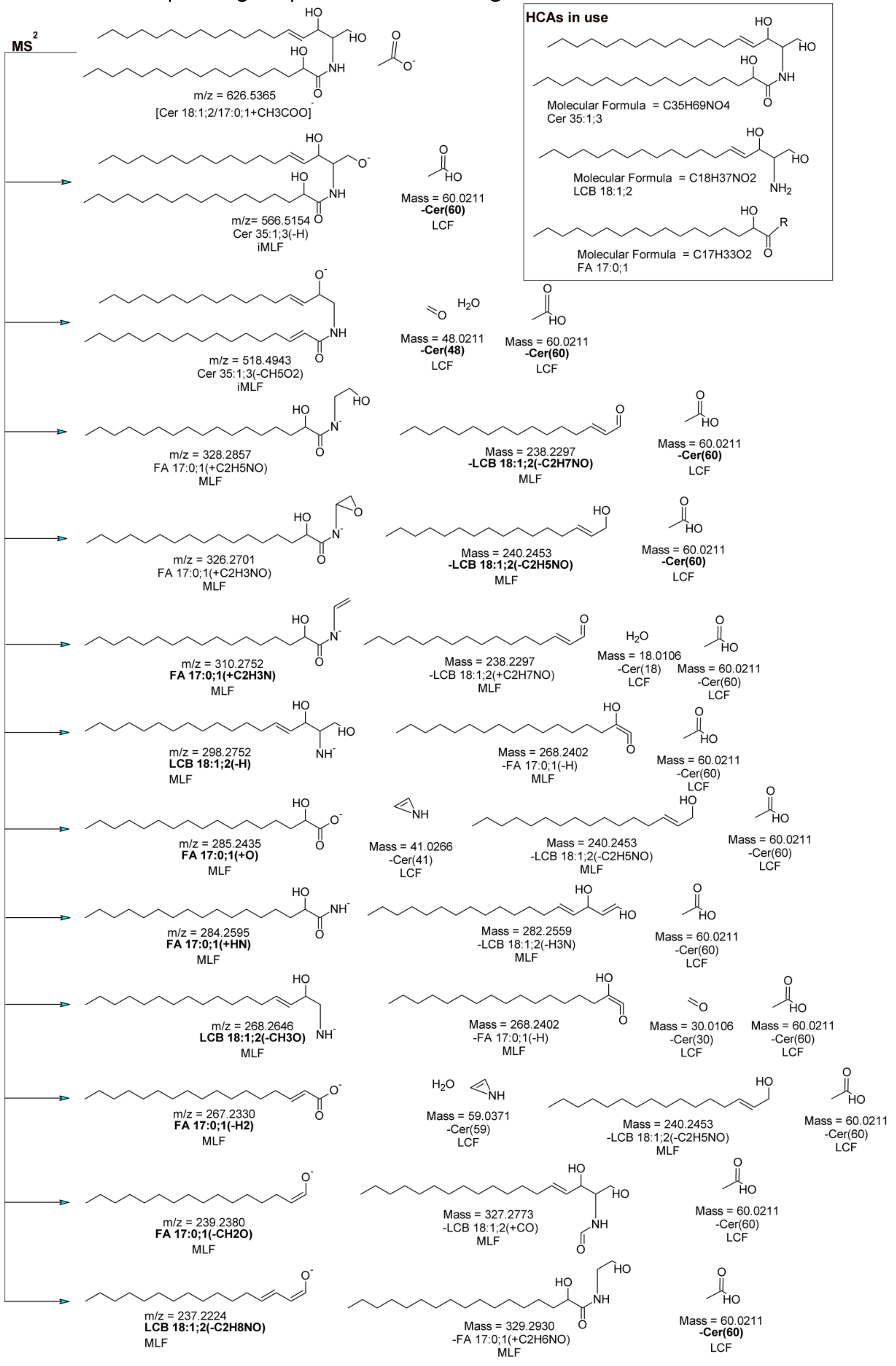

S3H Fig) SE 27:1/19:0

Proposed structures corresponding to spectrum shown in Fig. 3H.

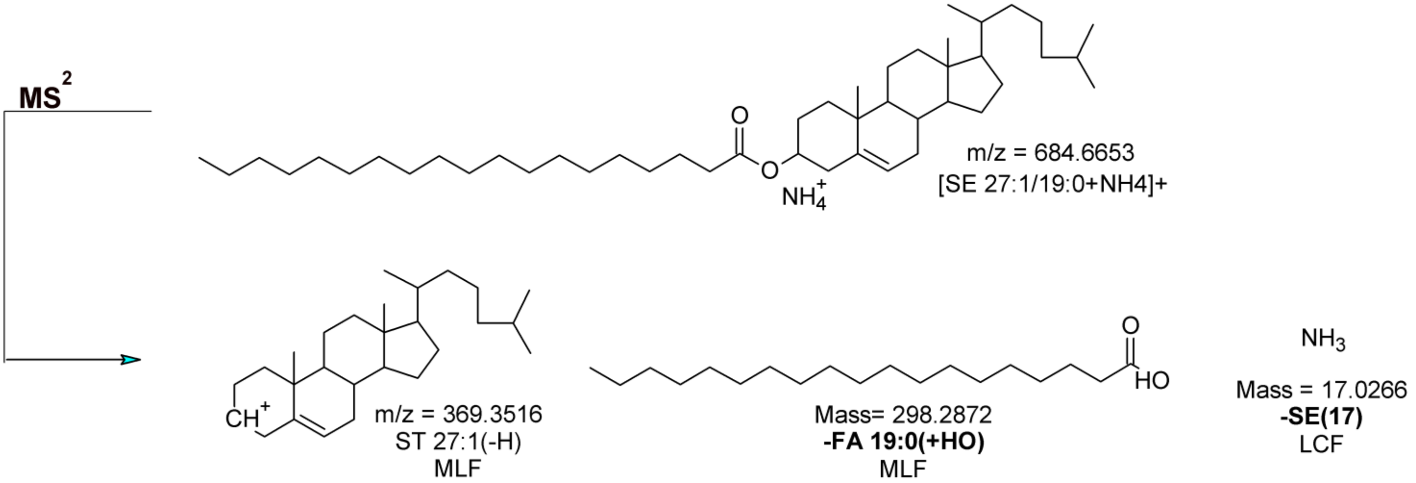

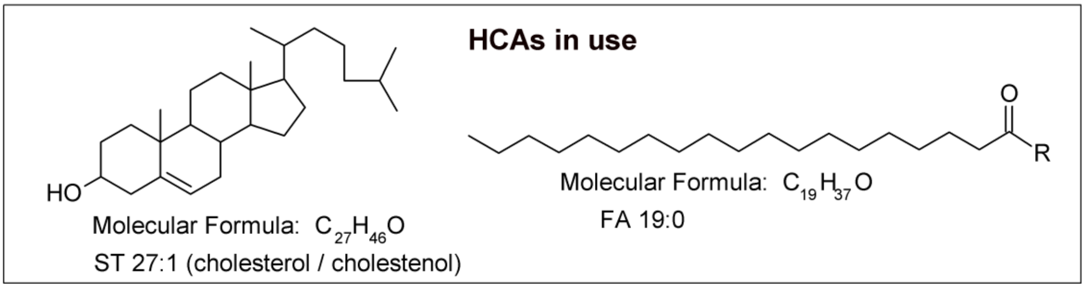

Supplement: S3 Fig — (PDF) [file pone.0188394.s005.pdf]
