# Supplementary material for: Proposal for a common nomenclature for fragment ions in mass spectra of lipids
Source: PLoS One. 2017 Nov 21;12(11):e0188394. doi: 10.1371/journal.pone.0188394 (PMC5697860; doi:10.1371/journal.pone.0188394)
Supplement: S4 Fig — (PDF) [file pone.0188394.s006.pdf]

S4 Fig

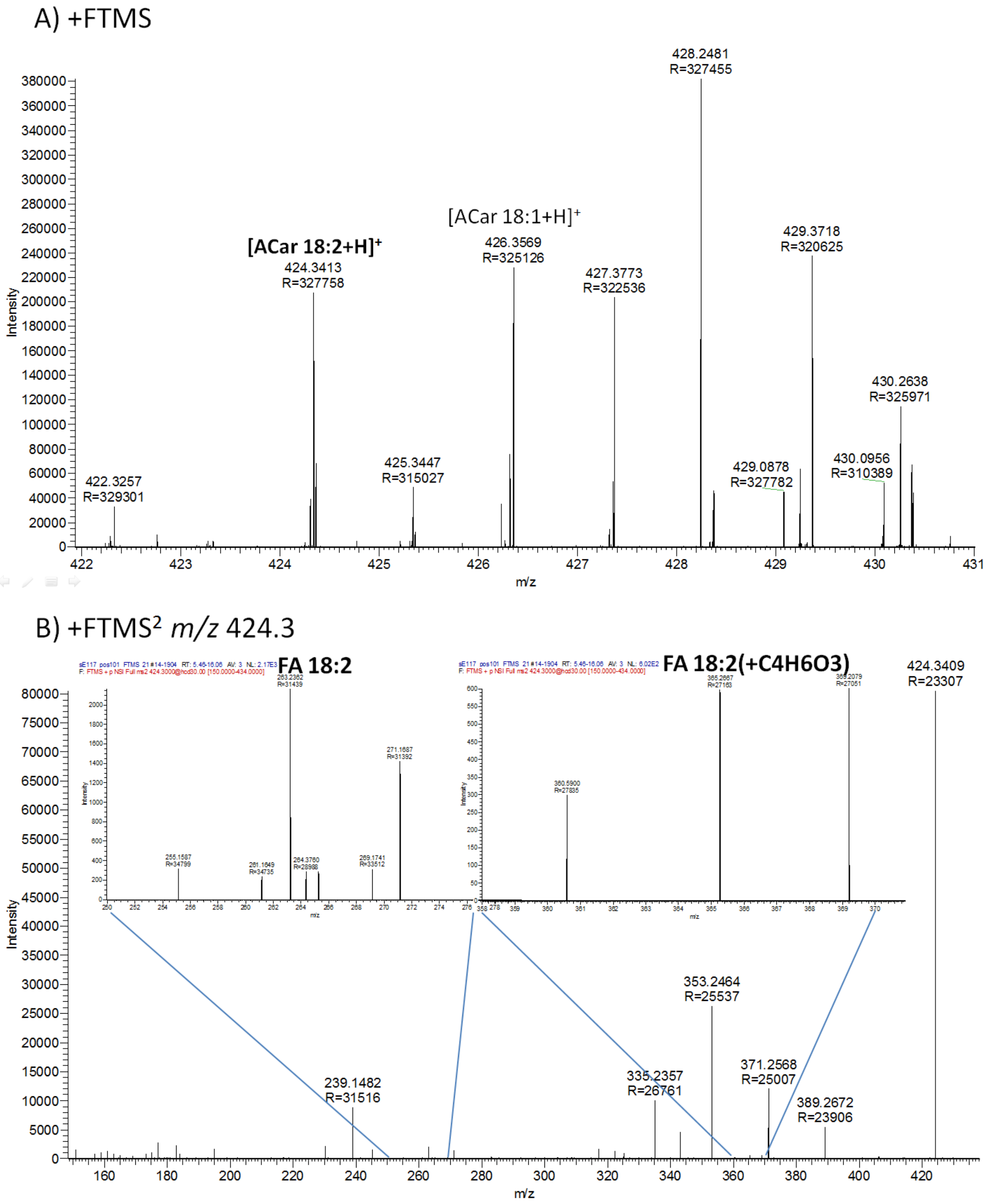

Fig. S4. Identification of low abundance ACar 18:2 in mouse plasma. A) Positive FTMS spectrum mouse plasma. The precursor ion matching protonated ACar 18:2 is highlighted in boldface. B) Positive $FTMS^2$ spectrum of *m/z* 424.3 with detection of MLFs matching ACar 18:2, annotated in boldface.
