# Supplementary material for: Proposal for a common nomenclature for fragment ions in mass spectra of lipids
Source: PLoS One. 2017 Nov 21;12(11):e0188394. doi: 10.1371/journal.pone.0188394 (PMC5697860; doi:10.1371/journal.pone.0188394)
Supplement: S5 Fig — (PDF) [file pone.0188394.s007.pdf]

S5 Fig

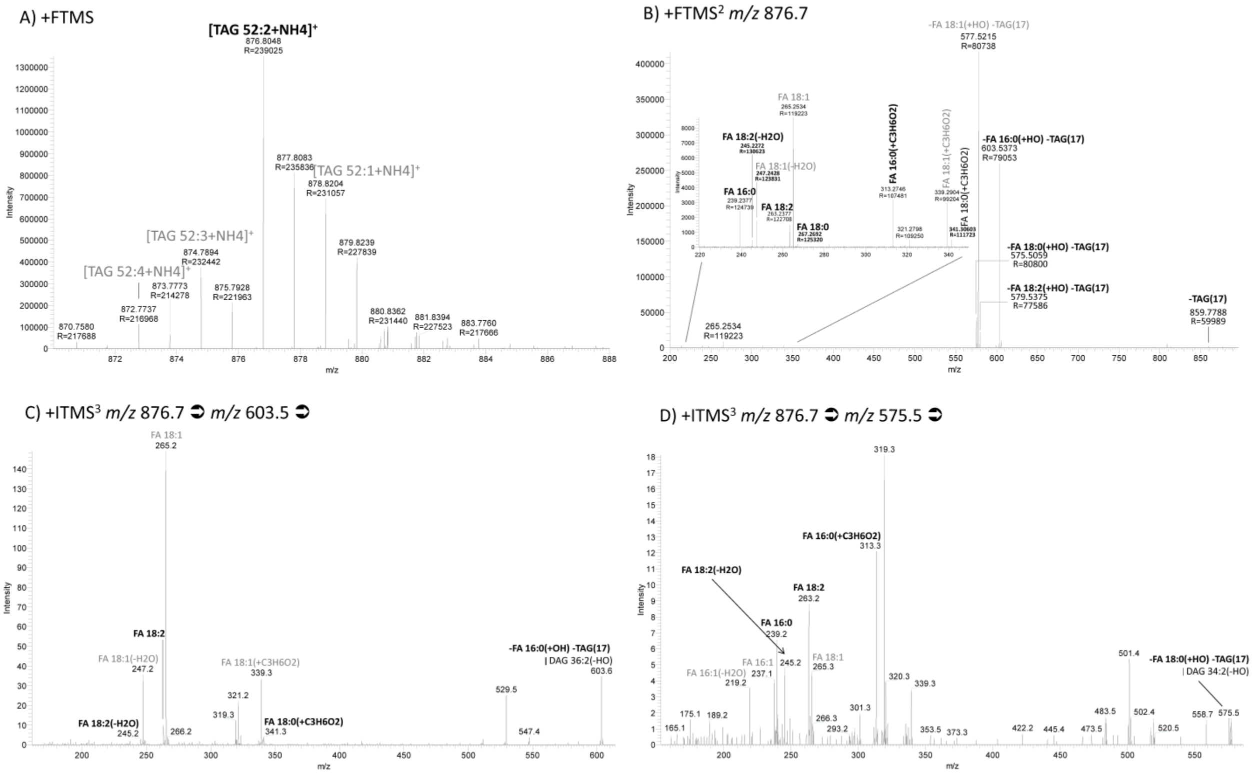

S5 Fig. Identification of low abundance TAG 16:0-18:0-18:2 in bovine liver. A) Positive FTMS spectrum of bovine liver. The precursor ion matching ammoniated TAG 52:2 is highlighted in boldface. B) Positive FTMS^2 spectrum of *m/z* 876.7. C) Positive ITMS^3 spectrum of *m/z* 603.5. D) Positive ITMS^3 spectrum of *m/z* 575.5. In all FTMS^2 and ITMS^3 spectra, MLFs matching TAG 16:0-18:0-18:2 are annotated in boldface whereas MLFs matching the more abundant and isomeric TAG 16:0-18:1-18:1 molecule are annotated in grey, except for 16:0-specific fragments which are shared.
