# Supplementary material for: Proposal for a common nomenclature for fragment ions in mass spectra of lipids
Source: PLoS One. 2017 Nov 21;12(11):e0188394. doi: 10.1371/journal.pone.0188394 (PMC5697860; doi:10.1371/journal.pone.0188394)

S6A Fig.

Proposed structures corresponding to spectrum shown in Fig. 6A

+FTMS^2 *m/z* 771.6, PC 34:2(+[2]H13)

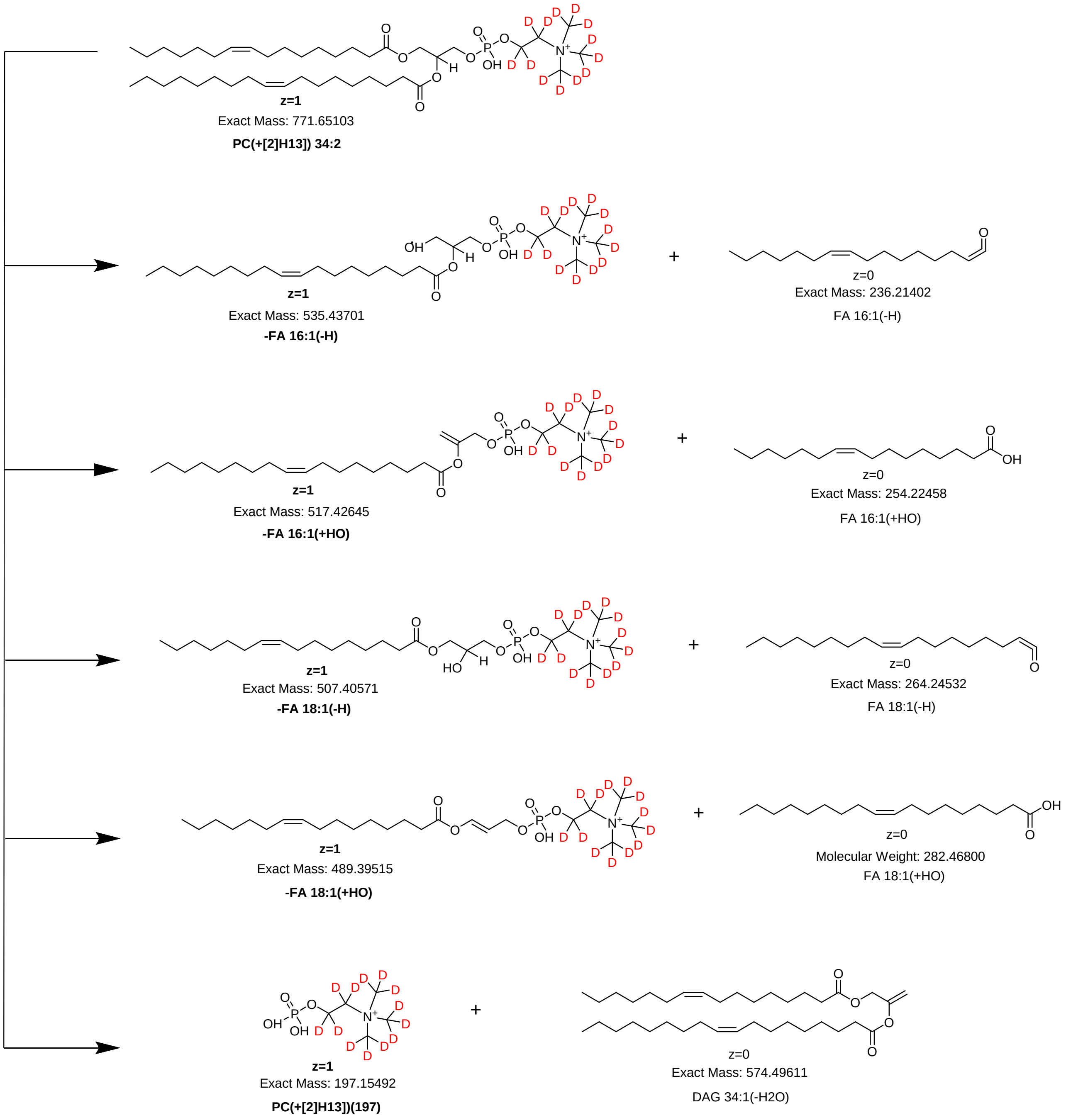

S6B Fig.

Proposed structures corresponding to spectrum shown in Fig. 6B.

+FTMS^2 *m/z* 740.6, PC 32:0(+[2]H6)

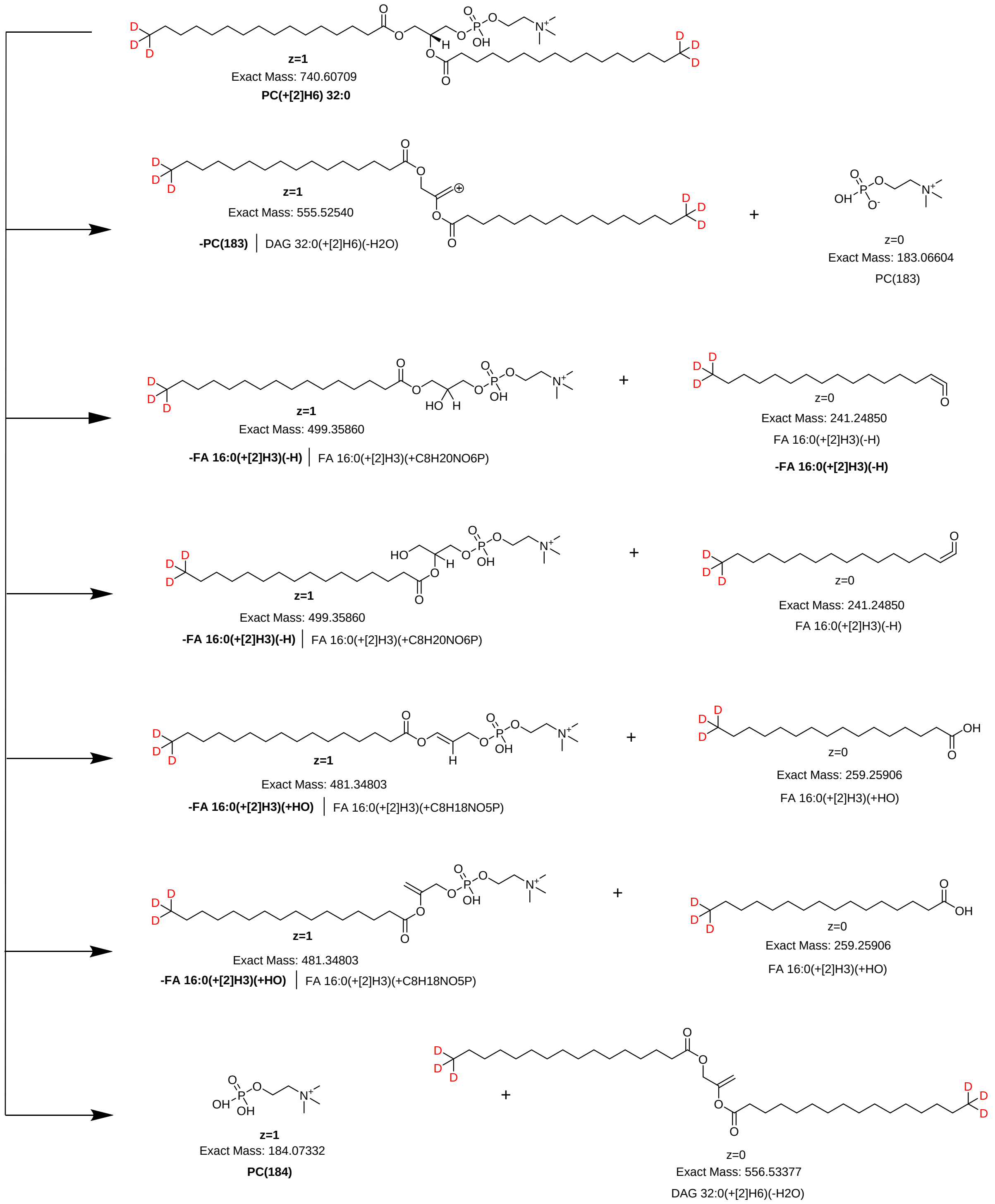

S6C Fig.

Proposed structures corresponding to spectrum shown in Fig. 6C.

-FTMS^2 *m/z* 841.6, PI 34:1(+[2]H6)

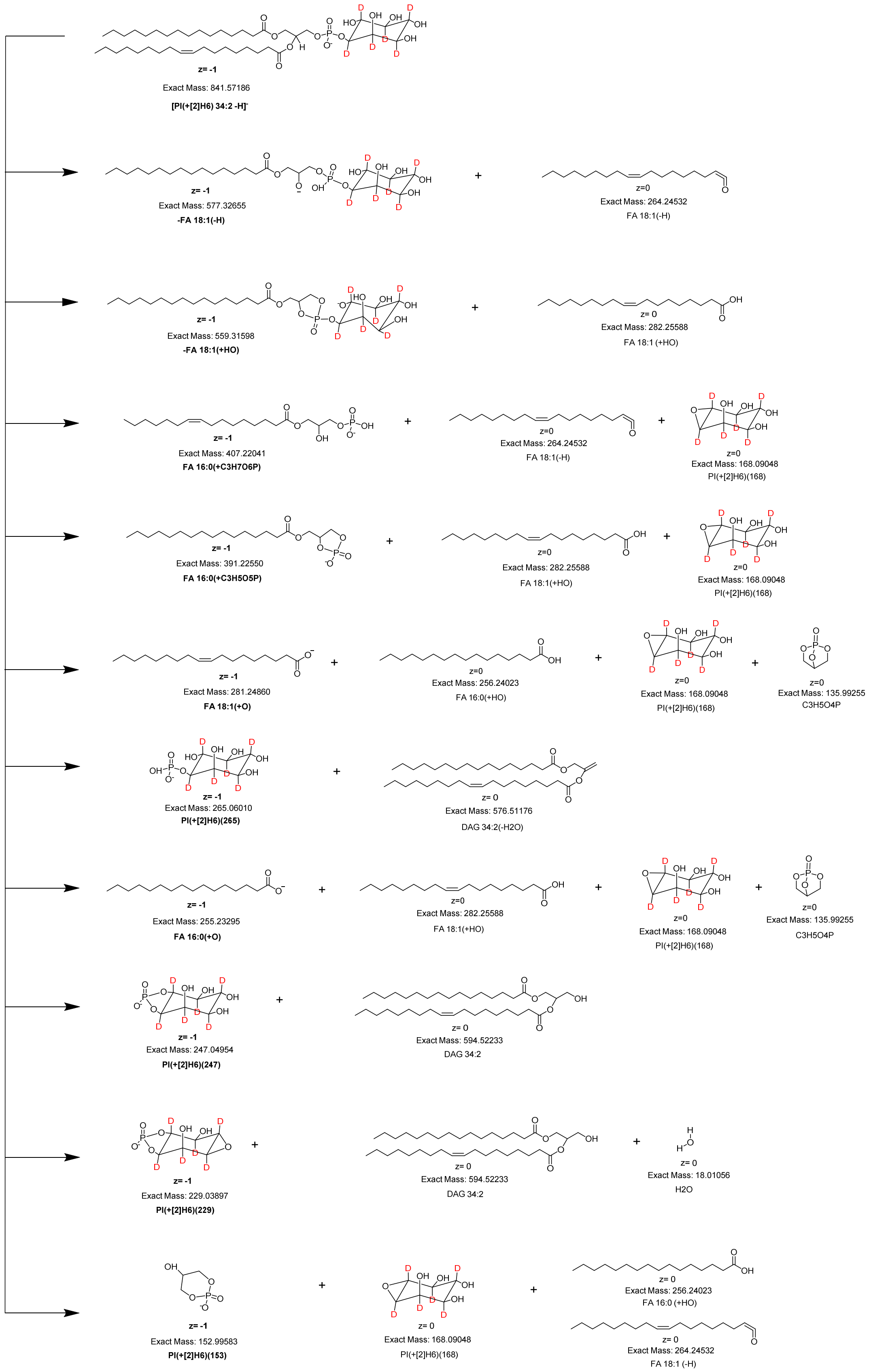

S6D Fig.

Proposed structures corresponding to spectrum shown in Fig. 6D.

-FTMS^2 *m/z* 759.7, Cer 44:0;4(+[13]C2[15]N)

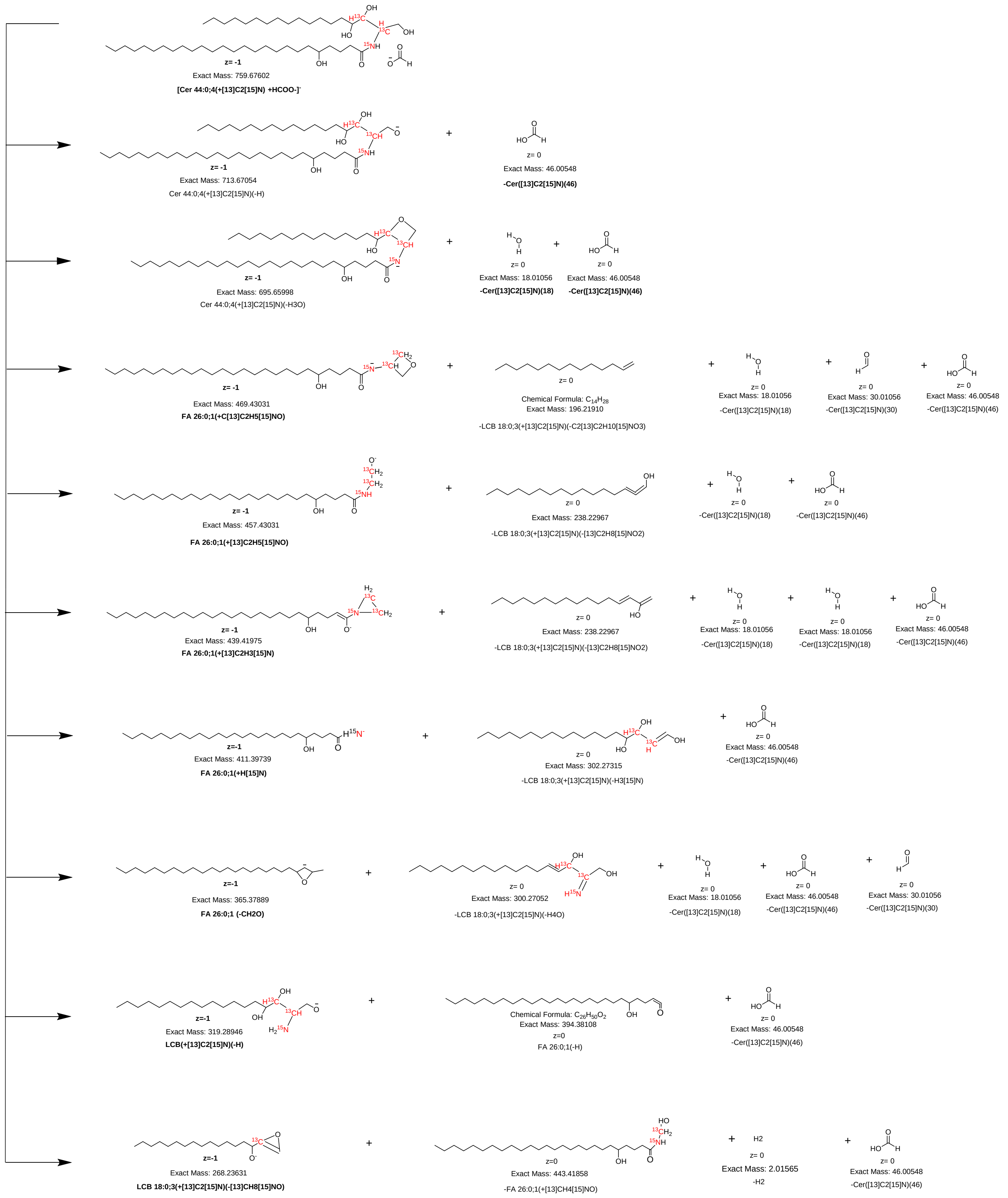

Supplement: S6 Fig — (PDF) [file pone.0188394.s008.pdf]
